# Supplementary material for: The decoy oligodeoxynucleotide against HIF-1α and STAT5 ameliorates atopic dermatitis-like mouse model
Source: Mol Ther Nucleic Acids. 2023 Sep 20;34:102036. doi: 10.1016/j.omtn.2023.102036 (PMC10550406; doi:10.1016/j.omtn.2023.102036)
Supplement: Document S1. Figures S1–S3 [file mmc1.pdf]

OMTN, Volume *34*

Supplemental information

The decoy oligodeoxynucleotide against HIF-1α and STAT5 ameliorates atopic dermatitis-like mouse model

Mi-Gyeong Gwon, Jaechan Leem, Hyun-Jin An, Hyemin Gu, Seongjae Bae, Jong Hyun Kim, and Kwan-Kyu Park

Supplemental Information

Figure S1

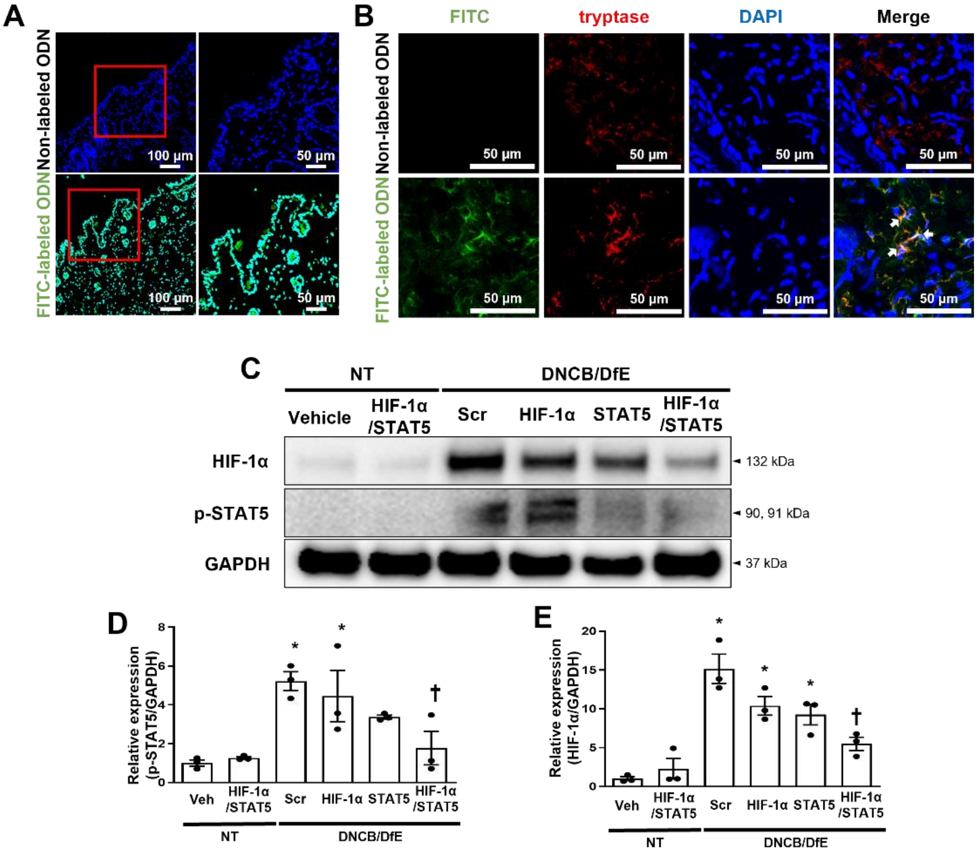

Figure S1. Introduction efficiency of the FITC-labeled HIF-1α/STAT5 decoy ODN in Balb/c mice and the inhibitory effect of the HIF-1α/STAT5 decoy ODN on HIF-1α and STAT5 expression.

(A) Fluorescence staining images show the transfection efficiency of the FITC-labeled HIF-1α/STAT5 decoy ODN (green) in the dorsal skin (n=2). The FITC-labeled decoy ODN was injected via the tail vein of the mice. The nuclei were stained with DAPI (blue). The right image of each group is an enlargement of the area marked with a red square. Scale bar = 100 or 50 μm. (B) Representative immunofluorescence staining image shows that the HIF-1α/STAT5 decoy ODN effectively enters mast cells in mouse skin (n=3). Green color indicates FITC-labeled decoy ODN, and red color indicates tryptase. The yellow signal in the merge panel is the co-localization of FITC-labeled decoy ODN and tryptase. The arrows indicate mast cells that show a double-positive signal of FITC-labeled decoy ODN and tryptase. (C) The protein expressions of HIF-1α and p-STAT5 were analyzed by immunoblotting. GAPDH was used to normalize the equal loading of all protein samples. The quantitative graphs show the protein expression of (D) HIF-1α and (E) p-STAT5 normalized with GAPDH. The bar graphs were quantified from three independent immunoblotting data. Vehicle (Veh): distilled water; Scrambled (Scr) ODN:

Scrambled decoy ODN; HIF-1α/STAT5 ODN: HIF-1α/STAT5 decoy ODN; NT: DNCB and DfE non-treated group; DNCB/DfE: DNCB and DfE sensitized group. *$p < 0.05$ compared with the vehicle group; †$p < 0.05$ compared with the DNCB/DfE-sensitized with Scr ODN group.

Figure S2

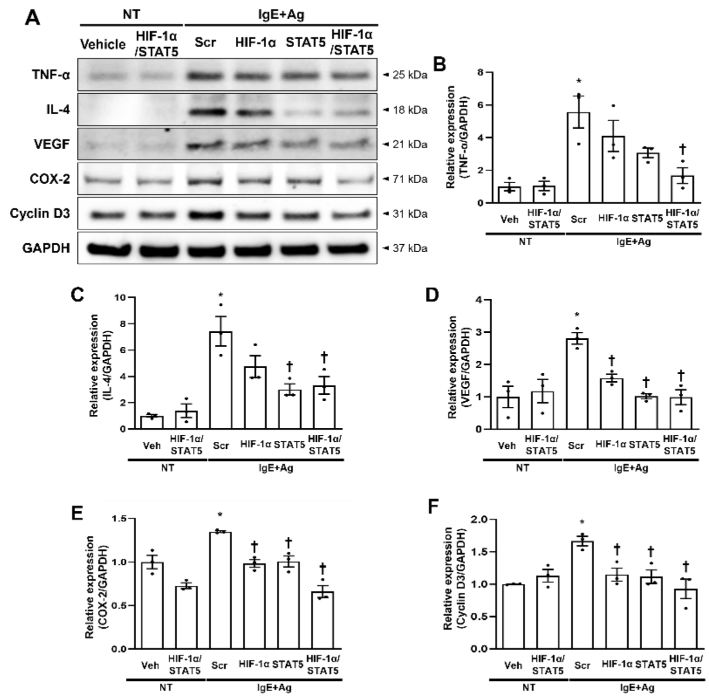

Figure S2. The inhibitory effect of the single decoy ODN and HIF-1α/STAT5 decoy ODN on HIF-1α and STAT5 downstream gene expression.

To investigate the effect of single decoy ODN, we designed two single decoy ODNs, a HIF-1α-scramble arm, and a STAT5-scramble arm. The RBL-2H3 cells were transfected with HIF-1α, STAT5, and HIF-1α/STAT5 decoy ODN (60 nM). Western blot analysis of cytokines, HIF-1α downstream genes (VEGF, COX-2), and STAT5 downstream gene (cyclin D3). GAPDH was used as a loading control. Quantification of immunoblots for (B) TNF-α, (C) IL-4, (D) VEGF, (E) COX-2, and (F) cyclin D3 after normalization with GAPDH. The graphs were quantified from three immunoblot data. Vehicle (Veh): distilled water; Scrambled (Scr) ODN: Scrambled decoy ODN; HIF-1α/STAT5 ODN: HIF-1α/STAT5 decoy ODN; NT: IgE+Ag non-treated; IgE+Ag: IgE+Ag treated. *$p < 0.05$ compared with the vehicle group; †$p < 0.05$ compared with the IgE+Ag-sensitized with Scr ODN group.

Figure S3

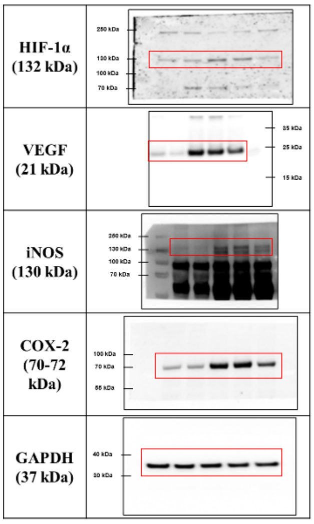

Figure 2C WB

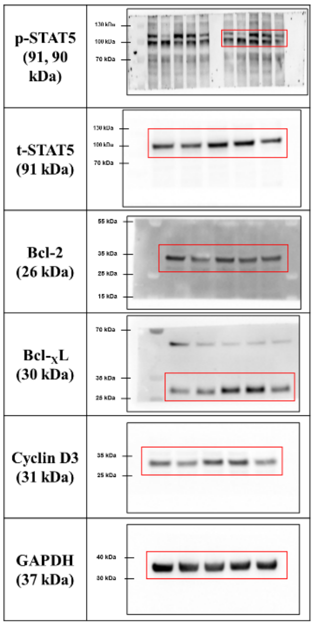

Figure 2H WB

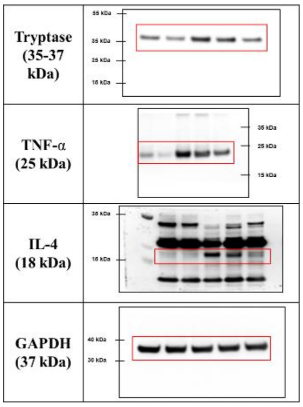

Figure 3C WB

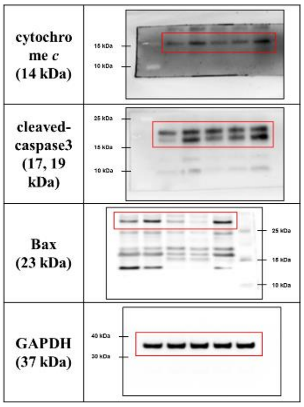

Figure 4B WB

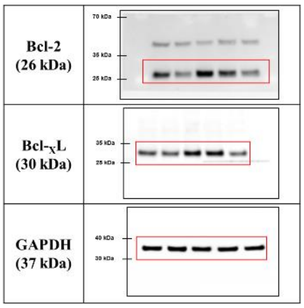

Figure 4F WB

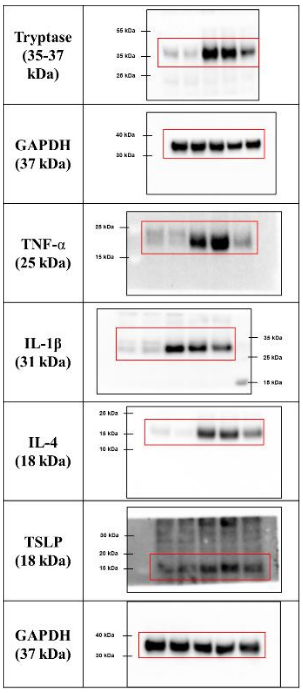

Figure 6C and 6E WB

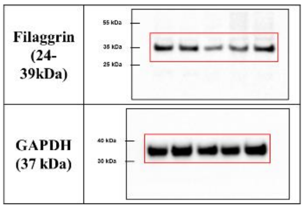

Figure 6L WB

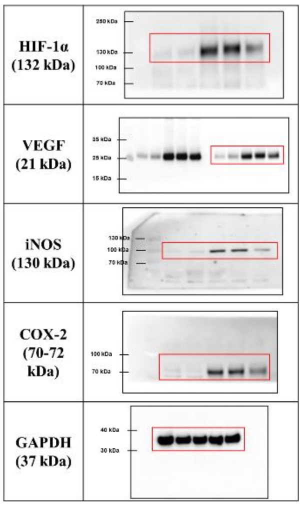

Figure 7C WB

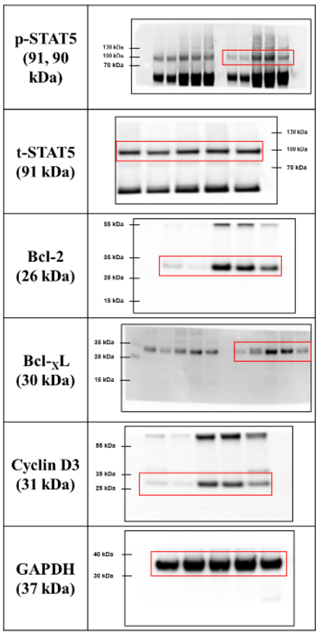

Figure 7H WB

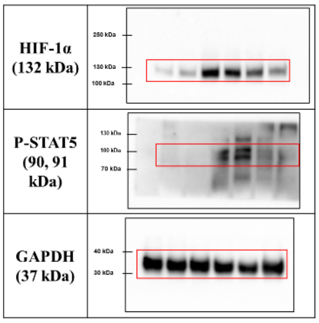

Figure S1C WB

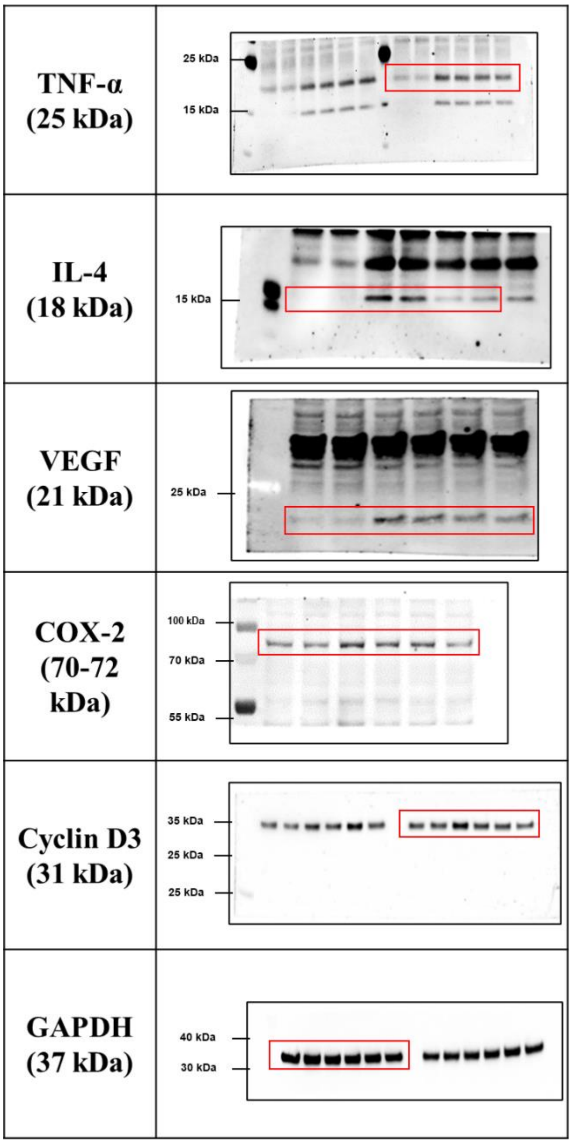

Figure S2A WB

Figure S3. Uncropped Western blot images from the blot are shown in all Figures.

The red boxes outline the areas presented in all the figures.
