# Supplementary material for: The decoy oligodeoxynucleotide against HIF-1α and STAT5 ameliorates atopic dermatitis-like mouse model
Source: Mol Ther Nucleic Acids. 2023 Sep 20;34:102036. doi: 10.1016/j.omtn.2023.102036 (PMC10550406; doi:10.1016/j.omtn.2023.102036)
Supplement: Document S2. Article plus supplemental information [file mmc2.pdf]

Molecular Therapy
Nucleic Acids
Original Article

The decoy oligodeoxynucleotide against HIF-1α and STAT5 ameliorates atopic dermatitis-like mouse model

Mi-Gyeong Gwon,[1,4] Jaechan Leem,[2,4] Hyun-Jin An,[1] Hyemin Gu,[1] Seongjae Bae,[1] Jong Hyun Kim,[3] and Kwan-Kyu Park[1]

[1]Department of Pathology, School of Medicine, Daegu Catholic University, Daegu 42472, Republic of Korea; [2]Department of Immunology, School of Medicine, Daegu Catholic University, Daegu 42472, Republic of Korea; [3]Department of Biochemistry, School of Medicine, Daegu Catholic University, Daegu 42472, Republic of Korea

Atopic dermatitis (AD) is a common inflammatory skin disease caused by an immune disorder. Mast cells are known to be activated and granulated to maintain an allergic reaction, including rhinitis, asthma, and AD. Although hypoxia-inducible factor-1 alpha (HIF-1α) and signal transducer and activator of transcription 5 (STAT5) play crucial roles in mast cell survival and granulation, their effects need to be clarified in allergic disorders. Thus, we designed decoy oligodeoxynucleotide (ODN) synthetic DNA, without open ends, containing complementary sequences for HIF-1α and STAT5 to suppress the transcriptional activities of HIF-1α and STAT5. In this study, we demonstrated the effects of HIF-1α/STAT5 ODN using AD-like *in vivo* and *in vitro* models. The HIF-1α/STAT5 decoy ODN significantly alleviated cutaneous symptoms similar to AD, including morphology changes, immune cell infiltration, skin barrier dysfunction, and inflammatory response. In the AD model, it also inhibited mast cell infiltration and degranulation in skin tissue. These results suggest that the HIF-1α/STAT5 decoy ODN ameliorates the AD-like disorder and immunoglobulin E (IgE)-induced mast cell activation by disrupting HIF-1α/STAT5 signaling pathways. Taken together, these findings suggest the possibility of HIF-1α/STAT5 as therapeutic targets and their decoy ODN as a potential therapeutic tool for AD.

INTRODUCTION

Atopic dermatitis (AD) is a common chronic inflammatory and allergic skin disease affecting 1%–3% of adults and up to 15%–20% of children worldwide.[1] Furthermore, the incidence and severity of AD have increased 2- to 3-fold over the past several decades and have been steadily increasing to date.[2–4] The clinical symptoms of AD include erythema, scarring/dryness, skin thickening, and eczematous skin lesions, which are often accompanied by obvious itching and can have a significant impact on quality of life.[5–7]

Although the etiology of AD is not fully understood, numerous studies have suggested that AD occurs and worsens as a result of the complex interaction of multiple factors, including environmental, genetic, and immunological factors.[5,8,9] Immunologically, the primary pathogenic mechanism associated with the development of AD is an imbalance in T helper (Th) cells with allergy sensitization and the development of an inflammatory response to a specific allergen.[10] In the acute phase of AD, an abnormal Th2 cell immune response induces elevated levels of immunoglobulin E (IgE) and infiltration of AD effector cells, thereby potentially aggravating the disease and triggering a systemic Th2 cell response.[11,12] In addition, Th2-associated inflammatory cytokines and chemokines, including interleukin (IL)-4, IL-5, and IL-13, have a direct effect on skin cells, such as keratinocytes and mast cells.[2,13,14]

Mast cells are hemopoietic cells that reside in various tissues and are crucial components of innate and acquired immunity.[15,16] Furthermore, mast cells play an important role in type 2 immune responses and allergic inflammation diseases, such as AD and psoriasis.[17,18] Mast cells express the high-affinity IgE receptor (FcεRI) on their surfaces, which recognizes IgE and leads to cell activation upon cross-linking with allergens.[19] After activation, mast cells release allergic inflammatory mediators, such as serotonin, histamine, proteases, β-hexosaminidase, chemokines, and inflammatory cytokines, from secretory granules, resulting in a Th2-mediated allergic response.[20–22] Mast cells are capable of regulating the degranulation process and being reactivated, thereby inducing and sustaining the allergic response.[23] Moreover, the induction of mast cell apoptosis has been proposed as a potential therapeutic approach to treat allergic and chronic inflammatory diseases such as AD.[24,25]

FcεRI signaling is a well-known mechanism of mast cell activation that plays a crucial role in allergic inflammation and diseases.[26]

Received 2 April 2023; accepted 15 September 2023;
https://doi.org/10.1016/j.omtn.2023.102036.

[4]These authors contributed equally

Correspondence: Jong Hyun Kim, Department of Biochemistry, School of Medicine, Daegu Catholic University, Daegu 42472, Republic of Korea.
E-mail: kimjohn@cu.ac.kr
Correspondence: Kwan-Kyu Park, Department of Pathology, School of Medicine, Daegu Catholic University, Daegu 42472, Republic of Korea.
E-mail: kkpark@cu.ac.kr

This is an open access article under the CC BY license (http://creativecommons.org/licenses/by/4.0/).

This signaling is initiated by the crosslinking of multivalent antigens bound to IgE. When an allergen crosslinks with IgE, FcεRIs are phosphorylated by the Src family kinases, such as Lyn and Fyn, and initiate downstream cell signaling.[27,28] FcεRI-IgE-allergen complexes induce activation of the phosphatidylinositol 3-kinase, mitogen-activated protein kinase pathway, and various transcription factors, including nuclear factor κB (NF-κB), hypoxia-inducible factor 1 (HIF-1) complex, and signal transducer and activator of transcription (STAT).[29] Among transcription factors activated by FcεRI signaling, HIF-1α and STAT5 play an important role not only in allergic inflammation but also in mast cell activation and survival.

The HIF-1 complex is an important transcription factor for the cellular response to hypoxia and is a heterodimer composed of two subunits, namely, HIF-1α and HIF-1β.[30] Among these subunits, HIF-1α is an oxygen-regulated subunit whose stability affects HIF-1 transcriptional activity.[31] During hypoxic conditions, HIF-1α becomes stabilized and dimerized with HIF-1β to form the HIF-1 complex, which binds to the hypoxia response element (HRE) in the promoter region.[32] In addition to oxygen-dependent regulation, HIF-1α expression is controlled by various growth factors and cytokines through oxygen-independent pathways.[33] The HIF-1 complex regulates the expression of various target genes involved in several biological processes, such as angiogenesis, survival/apoptosis, cell adhesion/migration, activation of immune cells, and cytokine expression, which are crucial for inflammatory and innate immune responses.[33–35] In addition, several studies have demonstrated that HIF-1α stabilizes and accumulates in basophils and mast cells during allergic inflammation, and this accumulation occurs in an IgE-dependent manner.[36,37]

The pathogenesis of AD involves a complex interplay among immune dysregulation, genetic factors, and environmental triggers. The Janus kinase (JAK)-STAT signaling pathway has emerged as a key regulator in the development and progression of AD among the key immune response players.[38] JAK1 is a crucial enzyme involved in the signal transduction triggered by various cytokines and growth factors that contribute to the pathogenesis of AD.[39] In particular, IL-4, IL-13, and IL-31 have been identified as important cytokines driving the inflammatory response in AD.[40] In addition, emerging evidence suggests that STAT1 may play a role in the pathogenesis of this complex skin disease.[38] STAT1 can be activated by other cytokines, such as interferon (IFN), in addition to IL-4 and IL-13. Increased expression of IFN has been observed in AD lesions, suggesting that STAT1 signaling is involved in chronic inflammation and immune dysregulation in AD. Understanding the complex interactions among JAK1, STAT1, and other components of the JAK-STAT pathway in the pathogenesis of AD is critical for the development of targeted therapies.

The STAT family includes STAT1, STAT2, STAT3, STAT5A/B, and STAT6, which are intracellular transcription factors that regulate various aspects of cellular immunity, differentiation, proliferation, and apoptosis. In addition, several studies have revealed that STAT family members play a crucial role in allergic inflammation progression.[26,41] Among these STAT family members, STAT5 is an essential regulator of mast cell development and survival, as well as IgE-mediated functioning.[26] In mast cells, STAT5 is activated mainly by the stem cell factor receptor through JAK2 and FcεRI signaling through the Src family kinase. STAT5 dimers enter the nucleus upon activation and bind to the gamma-activated site (GAS) motifs in the promoter region to regulate the target genes, including Bcl-2, Bcl-$_X$L, and cyclin D3.[42,43] Taken together, the activation of HIF-1α and STAT5 contributes to the increase in the expression of pro-inflammatory mediators and the maintenance of mast cell homeostasis. Therefore, targeting HIF-1α and STAT5 may be a potential new therapeutic strategy to treat AD.

Decoy oligodeoxynucleotide (ODN), a type of gene therapy product, has been developed to block the activity of specific transcription factors. Decoy ODNs are synthetic DNA segments that are identical to the consensus sequence to which transcription factors can bind to express downstream genes. Decoy ODN subsequently binds to the target transcription factor and attenuates target gene expression by inhibiting the activity of transcription factors at the pre-transcription level.[44–46] Therefore, this decoy ODN strategy has been proposed as an effective therapeutic tool to treat several disorders by suppressing the expression of specific genes both *in vitro* and *in vivo*.[47–49] Our previous studies demonstrated the efficacy of synthetic decoy ODNs in various disease models, including renal fibrosis, liver fibrosis, and arteriosclerosis.[45,47,50] However, the therapeutic effect of HIF-1α/STAT5 decoy ODN in AD has not been demonstrated.

In this study, we evaluated the therapeutic effects of HIF-1α/STAT5 decoy ODNs, which contain a DNA-binding sequence without an open end, and block the expression of their downstream factors. We also demonstrated their therapeutic effects through validated diverse assays with AD *in vitro* and *in vivo* models.

RESULTS

Generation of HIF-1α/STAT5 decoy ODN

To investigate the effects of HIF-1α/STAT5 in AD, we generated a synthetic HIF-1α/STAT5 decoy ODN, which was designed as a double-stranded nucleotide containing the consensus binding sequences of HIF-1α (HRE; CACGT) and STAT5 (GAS; TTCCCGGAA). The HIF-1α/STAT5 decoy ODN was synthesized with a ring-type structure to prevent nuclease degradation and stabilize its expression. The two individual sequences corresponding to the binding motifs of HIF-1α and STAT5 had a small loop and a cohesive end, respectively (Figure 1A, top). These sequences were altered to a closed ODN with two small loops that could self-ligate with T4 DNA ligase enzymes (Figure 1A, bottom). The sequence of the HIF-1α/STAT5 decoy ODN do not match with any nucleotides except for the HIF-1α/STAT5 sequence, as determined using the Basic Local Alignment Search Tool program (data not shown). We used rat basophilic leukemia (RBL)-2H3 cells for our model system to study mast cell functions because bone-marrow-derived mast cells show low transfection efficiency.[51]

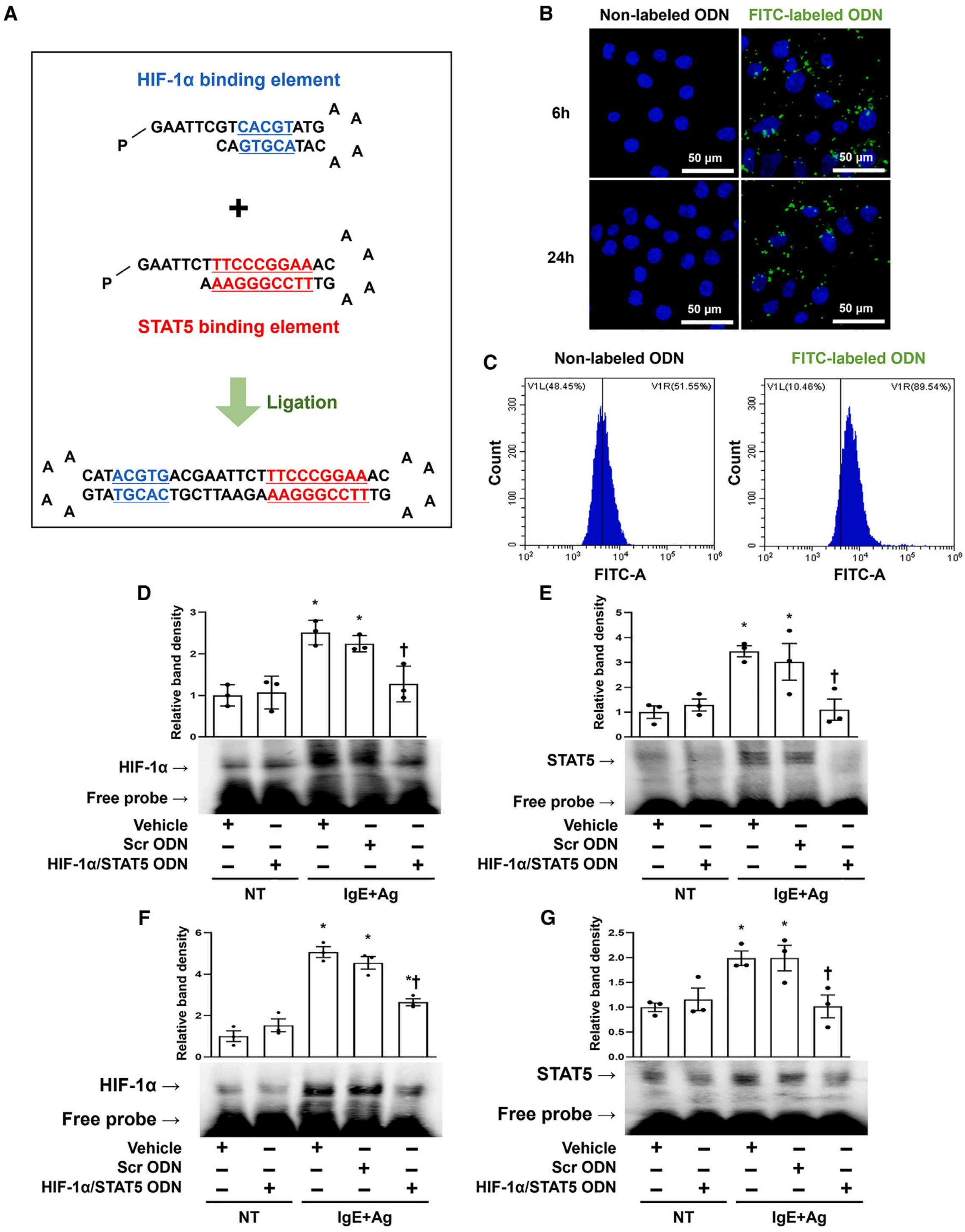

Figure 1. Structure of the synthetic HIF-1α/STAT5 decoy ODN and transfection efficiency of the HIF-1α/STAT5 decoy ODN in an AD-like *in vitro* and *in vivo* model

(A) Primary sequence of the HIF-1α/STAT5 decoy ODN. (B) The fluorescence result showed that HIF-1α/STAT5 decoy ODN was effectively transfected into RBL-2H3 cells. The RBL-2H3 cells were transfected with FITC-labeled HIF-1α/STAT5 decoy ODN (60 nM; green). The cells were then stained with DAPI (blue). Scale bar, 50 μm. (C) Transfection efficiency of the FITC-labeled HIF-1α/STAT5 decoy ODN was analyzed using flow cytometry. (D and E) HIF-1α- and STAT5 DNA-binding activity was analyzed using electrophoretic mobility shift assay (EMSA) using the nuclear extract of the RBL-2H3 cells. Three independent EMSA data were used to quantify the dot

(legend continued on next page)

HIF-1α/STAT5 decoy ODN suppressed HIF-1α and STAT5 DNA-binding activity in AD *in vitro* and *in vivo* models

To visualize the HIF-1α/STAT5 decoy ODN transfected into RBL-2H3 cells, we made a fluorescein isothiocyanate (FITC)-conjugated decoy ODN and analyzed the cellular distribution using confocal microscopy. FITC-labeled HIF-1α/STAT5 decoy ODN was found in both the cytoplasm and nucleus of the RBL-2H3 cells after 6 h transfection (Figure 1B, top). At 24 h after transfection, a green fluorescence signal was detected in both the cytoplasm and the nucleus (Figure 1B, bottom). Next, we used the liposome transfection method and then performed flow cytometry to count the transfection efficiency of the HIF-1α/STAT5 decoy ODN in RBL-2H3 cells. The green fluorescence signal in the RBL-2H3 cells transfected with the FITC-labeled decoy ODN had a value of 89.54%, showing a higher positive rate compared with the non-labeled decoy ODN (Figure 1C). Next, we verified the injection efficiency of the HIF-1α/STAT5 decoy ODN in mice before performing the animal experiments. The signal of FITC-labeled HIF-1α/STAT5 decoy ODN was observed in the skin when it was injected into the mouse through tail vein (Figure S1A). In addition, to determine whether HIF-1α/STAT5 decoy ODN is introduced into mast cells in mouse skin, we performed immunofluorescence using FITC-labeled decoy ODN and tryptase, a mast cell marker. As a result, we confirmed the part where FITC-labeled ODN and tryptase co-localize and show a yellow signal, indicating that HIF-1α/STAT5 decoy ODN enters the mast cell in mouse skin (Figure S1B). These results indicate that the HIF-1α/STAT5 decoy ODN is effectively transfected into RBL-2H3 cells and the dorsal skin of Balb/c mice.

After determining the efficacy of transfection, immunoblot analysis was performed to examine the effect of the HIF-1α, STAT5, and HIF-1α/STAT5 decoy ODNs in AD-like *in vitro* and *in vivo* models. The protein expression levels of HIF-1α, phospho-STAT5 (p-STAT5), and their downstream genes were reduced in each decoy ODN groups compared with the Scr decoy ODN group. Specifically, the HIF-1α/STAT5 decoy ODN attenuated these factor expression levels more significantly than the single decoy ODN. As a result, the HIF-1α/STAT5 decoy ODN significantly decreased cytokines, such as TNF-α and IL-4 (Figures S1C–S1E and S2). To investigate the cellular function of the HIF-1α/STAT5 decoy ODN, we performed an electrophoretic mobility shift assay (EMSA) with HIF-1α- or STAT5-binding oligonucleotide probes labeled with biotin. The DNA-binding activity of HIF-1α or STAT5 was increased in the IgE+Ag-treated RBL-2H3 cells compared with the non-treated cells. The HIF-1α-DNA or STAT5-DNA complexes were significantly decreased in the HIF-1α/STAT5 decoy ODN compared with the Scr ODN in IgE+Ag-treated RBL-2H3 cells (Figures 1D and 1E). Furthermore, the same results were confirmed in animal experiments. HIF-1α/STAT5 decoy ODN significantly inhibited the DNA-binding activity of HIF-1α and STAT5 compared with the AD-like skin disease mouse model (Figures 1F and 1G). These results indicate that the HIF-1α/STAT5 decoy ODN effectively prevents the DNA-binding activity of both HIF-1α and STAT5 at the transcription level.

HIF-1α/STAT5 decoy ODN suppressed the expression levels of HIF-1α and STAT5 and downstream factors in IgE+Ag-treated RBL-2H3 cells

We first measured HIF-1α and STAT5 expression levels after transfection with the HIF-1α/STAT5 decoy ODN in RBL-2H3 cells using immunofluorescent staining. The expression of HIF-1α and STAT5 was significantly increased in the IgE+Ag-treated RBL-2H3 cells compared with the normal cells. Their expression levels were significantly decreased in the RBL-2H3 cells transfected with the HIF-1α/STAT5 decoy ODN compared with the scrambled ODN (Figures 2A and 2B). We performed an immunoblot assay to examine the expression levels of HIF-1α, STAT5, and their downstream factors. We selected VEGF, iNOS, and COX-2 as downstream factors of HIF-1α and Bcl-2, Bcl-$_X$L, and cyclin D3 as downstream factors of STAT5 through a survey of several published reports.[42,52,53] The expression levels of HIF-1α and STAT5 were significantly attenuated in the RBL-2H3 cells transfected with the HIF-1α/STAT5 decoy ODN (Figures 2C, 2D, 2H, and 2I). The protein expression levels of VEGF, iNOS, and COX-2 were significantly attenuated in the RBL-2H3 cells transfected with the HIF-1α/STAT5 decoy ODN compared with the normal cells (Figures 2C, 2E, 2F, and 2G). The expression levels of Bcl-2, Bcl-$_X$L, and cyclin D3 were significantly attenuated in the RBL-2H3 cells transfected with the HIF-1α/STAT5 decoy ODN compared with the normal cells (Figures 2H, 2J, 2K, and 2L). Together, these results indicate that the HIF-1α/STAT5 decoy ODN suppressed the expression levels of HIF-1α, STAT5, and their downstream factors. Subsequently, we focused on the pro-inflammatory cytokine tryptase, which is used as an indicator of allergic response in RBL-2H3 cells. We first performed immunofluorescence staining, and immunofluorescent images were visualized using confocal microscopy. The tryptase that stained a green color was decreased in the IgE+Ag-treated RBL-2H3 cells transfected with the HIF-1α/STAT5 decoy ODN compared with the scrambled decoy ODN (Figures 3A and 3B). To further validate these results, we performed an immunoblot assay with tryptase, TNF-α, and IL-4 because mast cells are known to secrete the pro-inflammatory cytokines TNF-α, and IL-4 after stimulation with IgE+Ag.[54,55] The HIF-1α/STAT5 decoy ODN significantly attenuated the expression levels of tryptase, TNF-α, and IL-4 compared with the scrambled decoy ODN (Figures 3C–3F). These results indicate that the HIF-1α/STAT5 decoy ODN significantly inhibited the pro-inflammatory cytokines in the IgE+Ag-treated RBL-2H3 cells.

plots. +, treated; −, un-treated; vehicle (Veh), distilled water; scrambled (Scr) ODN, scrambled decoy ODN; HIF-1α/STAT5 ODN, HIF-1α/STAT5 decoy ODN; NT, IgE+Ag non-treated; IgE+Ag, IgE+Ag treated. *$p < 0.05$ compared with the vehicle group; †$p < 0.05$ compared with the IgE+Ag-sensitized group. (F and G) DNA-binding activity of HIF-1α and STAT5 was measured using EMSA using the mouse nuclear extract. The dot graphs were quantified from three independent EMSA data. +, treated; −, un-treated; vehicle (Veh), distilled water; scrambled (Scr) ODN, scrambled decoy ODN; HIF-1α/STAT5 ODN, HIF-1α/STAT5 decoy ODN; NT, DNCB and DfE non-treated group; DNCB/DfE, DNCB- and DfE-sensitized group. *$p < 0.05$ compared with the vehicle group; †$p < 0.05$ compared with the DNCB/DfE-sensitized group.

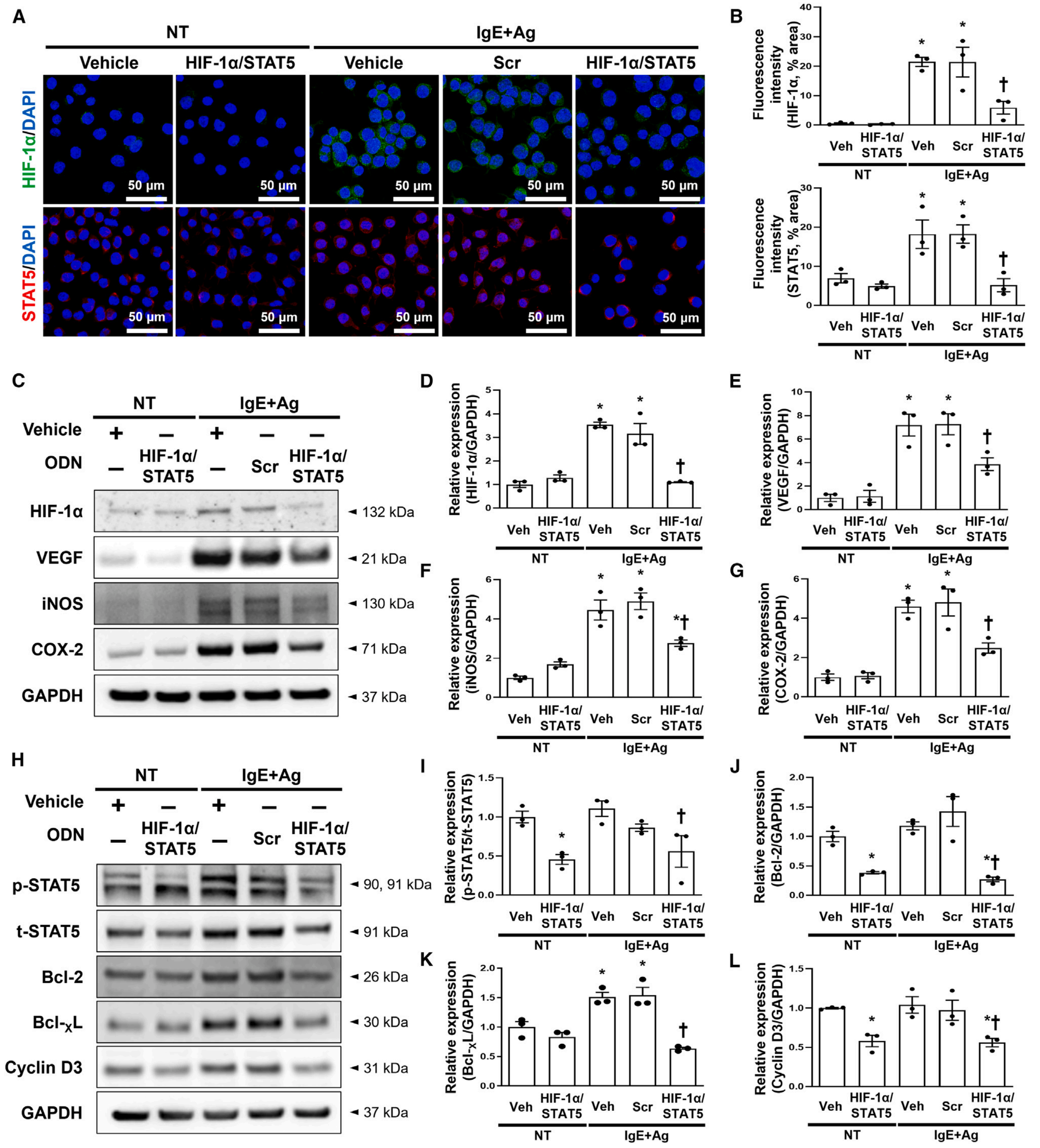

Figure 2. The HIF-1α/STAT5 decoy ODN significantly suppressed the expression of HIF-1α and STAT5 and attenuated their downstream target genes in IgE+Ag-sensitized RBL-2H3 cells

(A) Representative images of the immunofluorescence staining for HIF-1α (green, top) and STAT5 (red, bottom) in RBL-2H3 cells. The nuclei were labeled with DAPI (blue). Scale bar, 50 μm. (B) Quantification of the HIF-1α and STAT5 immunofluorescence signals. (C) Western blot analysis of HIF-1α and HIF-1α-related genes, including VEGF,

(legend continued on next page)

HIF-1α/STAT5 decoy ODN inhibited mast cell survival via the regulation of apoptosis signaling

Because several studies have shown that STAT5 is a critical transcription factor for mast cell survival and proliferation and that the HIF-1 transcription complex plays a crucial role in the adaption of various immune cells to hypoxic and inflammatory stresse,[56–58] we investigated whether HIF-1α and STAT5 transcription factors can induce mast cell apoptosis or not. We performed a TUNEL assay, which is an apoptosis monitoring assay, using the IgE+Ag-treated RBL-2H3 cells. The HIF-1α/STAT5 decoy ODN induced apoptosis in IgE+Ag-treated RBL-2H3 compared with normal cells (Figure 4A). Next, under the same conditions, we examined the expression levels of apoptosis-related proteins, such as cytochrome *c*, cleaved caspase-3, Bax, Bcl-2, and Bcl-$_X$L. Expression of the pro-apoptotic proteins Bax, cytochrome *c*, and cleaved caspase-3 was substantially increased in the RBL-2H3 cells treated with the HIF-1α/STAT5 decoy ODN, which was consistent with the TUNEL results (Figures 4B–4E). In contrast, expression of the anti-apoptotic proteins Bcl-2 and Bcl-$_X$L was decreased in the RBL-2H3 cells treated with the HIF-1α/STAT5 decoy ODN (Figures 4F–4H). These results indicate that the HIF-1α/STAT5 decoy ODN can induce apoptosis of RBL-2H3 cells.

HIF-1α/STAT5 decoy ODN attenuated the morphological changes in AD-like skin lesions in a 1-chloro-2,4-dinitrobenzene/*Dermatophagoides farinae* extract-induced mouse model

On the basis of the results of the *in vitro* observations for AD, we performed various experiments to examine the therapeutic effect of the HIF-1α/STAT5 decoy ODN in an AD-like skin disease animal model. *D. farinae* is a type of house dust mite that is a common environmental allergen associated with AD. 1-chloro-2,4-dinitrobenzene (DNCB) and *D. farinae* extract (DfE) are routinely used to experimentally induce AD by inducing both acute and chronic AD lesions.[59–61] We used an AD-like mouse model induced by DNCB/DfE to validate the effect of the HIF-1α/STAT5 decoy ODN on AD. The experimental procedure of AD-like skin disorder induction is schematically described in Figure 5A. Macroscopically, the dorsal skin lesions in the DNCB- and DfE-sensitized mice showed considerably increased physical signs of AD, such as erythema, punctiform, edema, hemorrhage, dryness, and crusting, compared with normal mice (Figure 5B). The administration of the HIF-1α/STAT5 decoy ODN alleviated AD-like skin lesions compared with the scrambled ODN. It was reported that AD skin has various characteristic clinical symptoms, such as redness, scabs, and keratosis, accompanied by inflammation and histopathological changes, including epidermal/dermal thickening and infiltration of immune cells within the lesions.[6] Thus, we performed H&E staining to determine the effect of the HIF-1α/STAT5 decoy ODN on DNCB/DfE-induced AD-like symptoms. The skin lesions of the DNCB/DfE-stimulated mice showed distinct hyperkeratosis, thickened epidermis and dermis, and immune cell infiltration in the dermis. The extent of skin thickness and immune cell infiltration was significantly decreased by injection with the HIF-1α/STAT5 decoy ODN compared with the scrambled ODN (Figures 5C–5E). These results imply that the HIF-1α/STAT5 decoy ODN may ameliorate the symptoms of AD-like skin lesions in the DNCB/DfE-sensitized Balb/c mice.

HIF-1α/STAT5 decoy ODN alleviated mast cell infiltration and degranulation in DNCB/DfE-induced AD-like skin disease

Mast cells are the principal effector cells of IgE-mediated allergic and inflammatory responses, which include common skin disorders such as AD and psoriasis.[24] In addition, IgE is involved in the development of allergic diseases, and the overproduction of serum IgE is a characteristic of AD.[2] We analyzed mast cell infiltration and degranulation using Giemsa staining and noted that mast cell infiltration and degranulation in AD-like skin lesions was significantly increased in the DNCB/DfE-induced AD-like mouse model. However, the administration of the HIF-1α/STAT5 decoy ODN significantly decreased mast cell infiltration and degranulation (Figures 5F–5I). HIF-1α/STAT5 decoy ODN administration also significantly decreased the levels of serum IgE, which were upregulated by the DNCB/DfE-induced AD-like mouse model, whereas the scrambled ODN decoy ODN did not affect serum IgE levels (Figure 5J). These results indicate that the HIF-1α/STAT5 decoy ODN ameliorates mast cell infiltration and degranulation in DNCB/DfE-induced AD-like skin disease.

HIF-1α/STAT5 decoy ODN suppressed DNCB/DfE-mediated pro-inflammatory cytokines

During degranulation, tryptase is released from mast cells, along with other granule products such as chymase and histamine.[62] Because tryptase is the most abundant mast cell secretory granule-derived serine proteinase,[63] we performed immunohistochemistry to detect tryptase after treatment with the HIF-1α/STAT5 decoy ODN in the DNCB/DfE-induced mice. Tryptase expression levels in the dorsal skin were markedly increased with the administration of the scrambled decoy ODN in the DNCB/DfE-induced mice, whereas its expression level was inhibited with administration of the HIF-1α/STAT5 decoy ODN (Figures 6A and 6B). We obtained similar results in the immunoblot, which showed that treatment of the HIF-1α/STAT5 decoy ODN inhibited the expression of tryptase in the tissue removed from the dorsal skin of DNCB/DfE-induced mice (Figures 6C and 6D). In addition, we investigated the expression level of pro-inflammatory cytokines such as TNF-α, IL-1β, IL-4, and thymic stromal lymphopoietin (TSLP). It was reported that TSLP, a novel cytokine, functions as a regulator in Th2-driven inflammatory diseases.[64] The protein expression of TNF-α, IL-1β, IL-4, and TSLP was increased in the DNCB/DfE-induced AD-like skin disease. However, administration of the HIF-1α/STAT5 decoy ODN drastically

iNOS, and COX-2. GAPDH was used as a loading control. (D–G) The dot graphs were quantified from three independent immunoblot data. (H) Immunoblot analysis of p-STAT5, t-STAT5, Bcl2, Bcl-$_X$L, and cyclin D3. GAPDH was used as a loading control. (I–L) Three independent immunoblots were used to quantify the dot graphs. +, treated; −, un-treated; vehicle (Veh), distilled water; scrambled (Scr) ODN, scrambled decoy ODN; HIF-1α/STAT5 ODN, HIF-1α/STAT5 decoy ODN; NT, IgE+Ag non-treated; IgE+Ag, IgE+Ag treated. *$p < 0.05$ compared with the vehicle group; $^{\dagger}p < 0.05$ compared with the IgE+Ag-sensitized group.

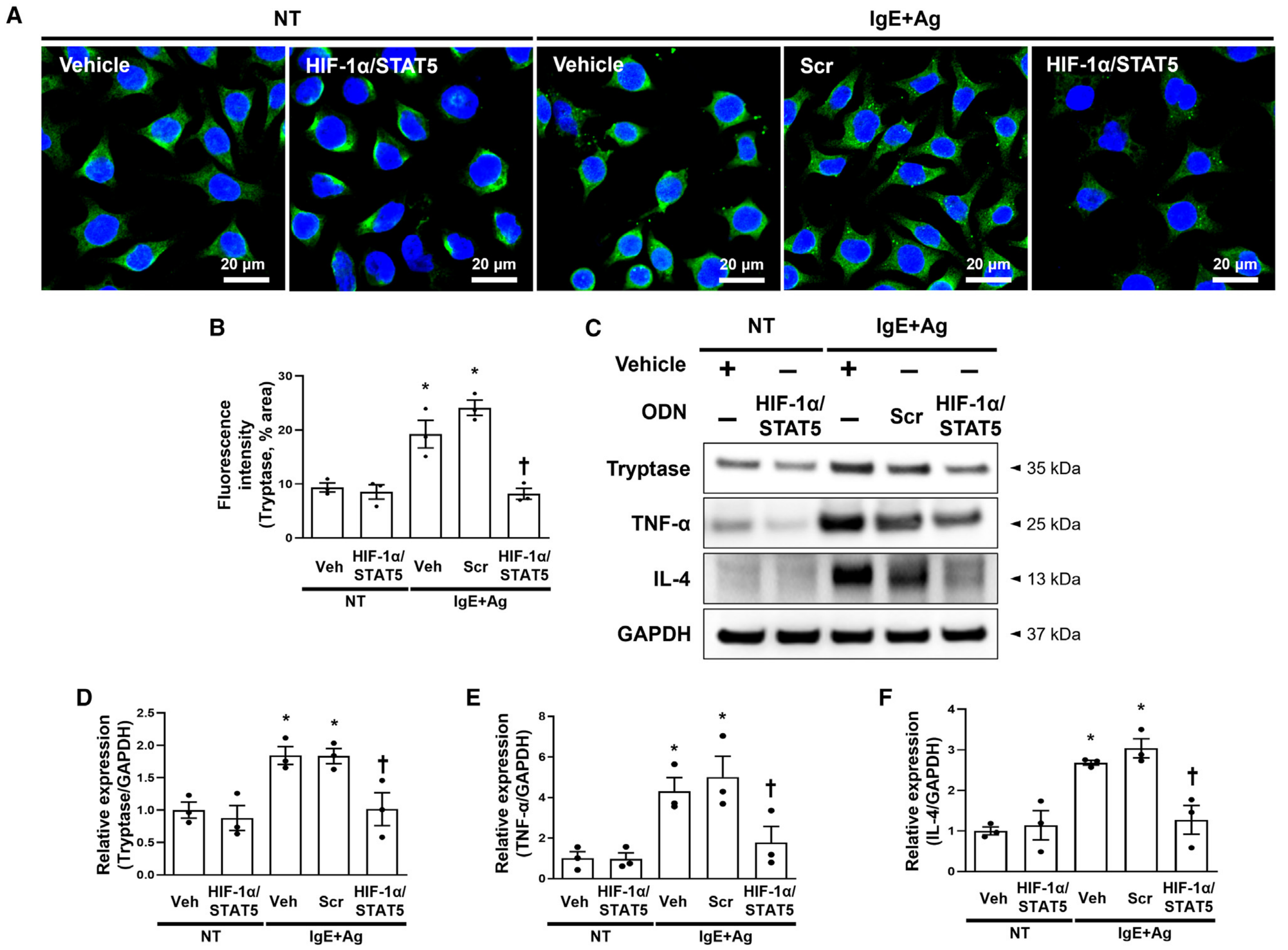

Figure 3. Effect of the HIF-1α/STAT5 decoy ODN on IgE-mediated degranulation in IgE+Ag-challenged RBL-2H3 cells

(A) Immunofluorescence staining for tryptase (green). The nuclei were labeled with DAPI (blue). Scale bar, 20 μm. (B) Quantification of the tryptase fluorescence signal. The dot graphs were quantified from three independent immunofluorescence images. (C) Immunoblot result showed that the HIF-1α/STAT5 decoy ODN attenuated the cytokine expression. GAPDH was used as a loading control. (D–F) The graphs show the quantification of (D) tryptase, (E) TNF-α, and (F) IL-4, each normalized to GAPDH. The graphs were quantified from three independent immunoblot data. +, treated; −, un-treated; vehicle (Veh), distilled water; scrambled (Scr) ODN, scrambled decoy ODN; HIF-1α/STAT5 ODN, HIF-1α/STAT5 decoy ODN; NT, IgE+Ag non-treated; IgE+Ag, IgE+Ag treated. *$p < 0.05$ compared with the vehicle group; †$p < 0.05$ compared with the IgE+Ag-sensitized group.

attenuated the expression levels of pro-inflammatory cytokines in DNCB/DfE-induced AD-like skin disease compared with the scrambled decoy ODN (Figures 6E–6I). These results indicate that the HIF-1α/STAT5 decoy ODN suppressed the DNCB/DfE-mediated pro-inflammatory cytokines.

Administration of HIF-1α/STAT5 decoy ODN may rescue the filaggrin expression suppressed by DNCB/DfE

Filaggrin-related disruption of skin barrier can be a primary cause of AD.[65] Because filaggrin is the main structural component of the stratum corneum and acts as the skin barrier,[66] we evaluated whether the HIF-1α/STAT5 decoy ODN may prevent the suppression of filaggrin expression by DNCB/DfE-induced AD-like skin disease. To evaluate the effect of the HIF-1α/STAT5 decoy ODN on filaggrin-related barrier dysfunction, we performed immunofluorescence and immunoblot. Filaggrin expression was decreased in the DNCB/DfE-treated group compared with that of non-treated group. However, administration of the HIF-1α/STAT5 decoy ODN significantly restored filaggrin expression compared with the scrambled ODN (Figures 6J–6M). These results indicate that HIF-1α/STAT5 decoy ODN might rescue filaggrin expression that is suppressed by DNCB/DfE-induced AD-like skin disease.

Administration of HIF-1α/STAT5 decoy ODN suppresses the downstream factors in DNCB/DfE-sensitized AD-like skin lesions

Next, we investigated the suppressive effect of the HIF-1α/STAT5 decoy ODN in the DNCB/DfE mice model by analyzing the target

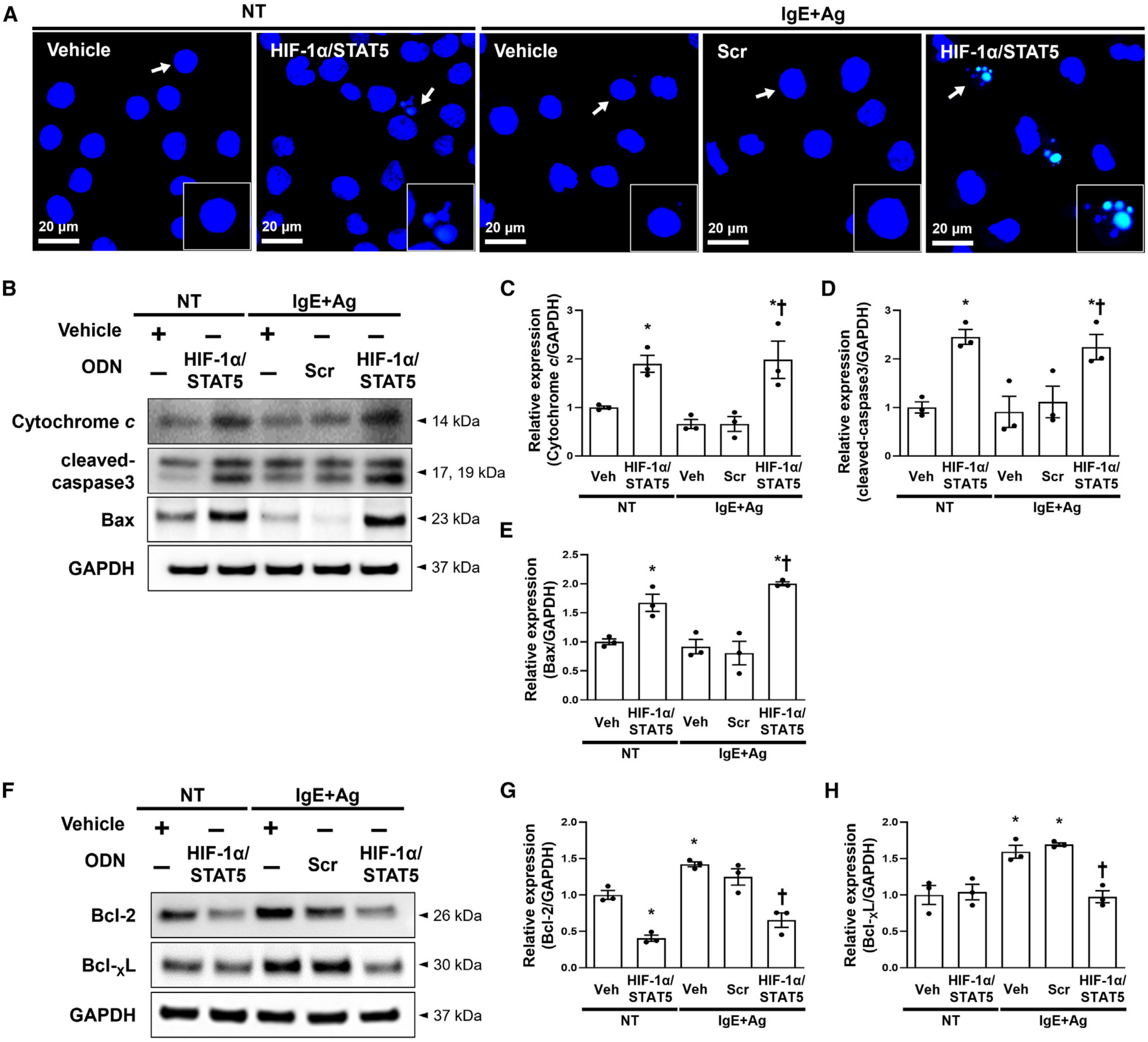

Figure 4. The HIF-1α/STAT5 decoy ODN-induced apoptosis of RBL-2H3 cells via the regulation of apoptosis-associated molecules

(A) TUNEL staining images of the RBL-2H3 cells from the indicated groups. DAPI was used to stain nuclei (blue). After TUNEL staining, these representative images were taken from random sites locations. Apoptotic cells are indicated by arrows, and representative images of apoptotic cells are indicated in the enlarged frames. Scale bar, 20 μm. (B) Immunoblotting analysis of pro-apoptotic proteins. GAPDH was used as a loading control. (C–E) The graphs show the quantification of (C) cytochrome *c*, (D) cleaved caspase-3, and (E) Bax, each normalized to GAPDH. The graphs were quantified from three independent immunoblot data. (F) Western blot analysis of anti-apoptotic proteins. All samples were loaded in equal volumes, as normalized by loading GAPDH together. (G and H) The graphs show the quantification of (G) Bcl-2 and (H) Bcl-$_X$L, each normalized to GAPDH. The dot graphs were quantified from three independent immunoblot data. +, treated; −, un-treated; vehicle (Veh), distilled water; scrambled (Scr) ODN, scrambled decoy ODN; HIF-1α/STAT5 ODN, HIF-1α/STAT5 decoy ODN; NT, IgE+Ag non-treated; IgE+Ag, IgE+Ag treated. *$p < 0.05$ compared with the vehicle group; $^{\dagger}p < 0.05$ compared with the IgE+Ag-sensitized group.

gene expression levels of the target genes via the transcriptional activities of HIF-1α and STAT5. Immunofluorescence showed the suppression of HIF-1α and STAT5 by administration of the HIF-1α/STAT5 decoy ODN in the DNCB/DfE-treated AD mice model (Figures 7A and 7B). Administration of the HIF-1α/STAT5 decoy ODN attenuated the expression of HIF-1α downstream genes, such as VEGF, iNOS, and COX-2, in the same model (Figures 7C–7G). It also inhibited the protein expression of STAT5 downstream genes, such as Bcl-2, Bcl-$_X$L, and cyclin D3, in the same model (Figures 7H–7L). Collectively, these results suggest that the HIF-1α/STAT5 decoy ODN affects the expression levels of downstream factors via transcriptional activities of HIF-1α and STAT5.

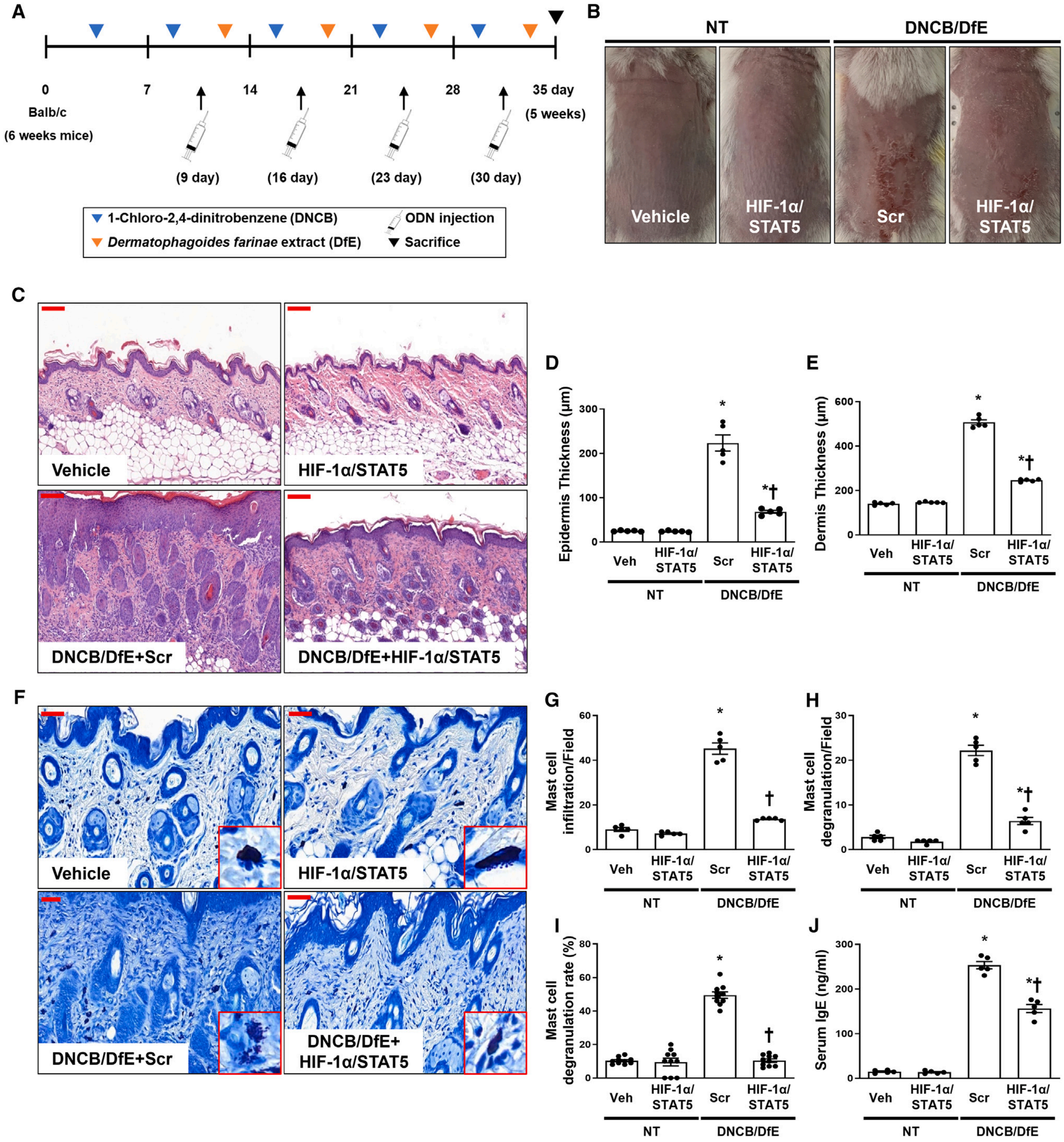

Figure 5. Effect of the HIF-1α/STAT5 decoy ODN in DNCB/DfE-induced AD-like symptoms, skin histological changes, and mast cell infiltration

(A) The schedule for induction of AD caused by DNCB/DfE sensitization and treatment of HIF-1α/STAT5 decoy ODN. (B) Balb/c mice dorsal skin lesions of each group. The animal experiment was performed in two independent experiments (n = 4 in the first experiment and n = 5 in the second experiment, for a total of n = 9 mice per group). (C) Paraffin-embedded sections of murine dorsal skin stained with hematoxylin and eosin. Scale bar, 100 μm. (D and E) The thicknesses of the (D) epidermis and (E) dermis were measured from at least 10 random fields per section at 200× magnification. (F) Paraffin-embedded sections of murine dorsal skin stained with Giemsa (n = 9). The representative images of mast cells are indicated in the enlarged frames. Scale bar, 50 μm. (G–I) The graphs show the statistical analysis of (G) the number of infiltrated mast

(legend continued on next page)

DISCUSSION

Various environmental and genetic factors are related to the development of allergic disease.[67] In recent years, the prevalence and severity of allergic disorders such as asthma, AD, eczema, and allergic rhinitis have increased significantly, and they have become an important public health problem affecting more than 300 million people worldwide.[68–70] Among these allergy-related diseases, AD is a chronic inflammatory skin disease that is characterized by allergic inflammatory responses and intense pruritus.[60] The therapeutic drugs mainly used for patients with AD are topical corticosteroids, anti-histamines, immunosuppressants, antibiotics, and calcineurin inhibitors (e.g., tacrolimus), which can reduce the inflammation and itching.[71] However, the long-term use of these currently available drugs can cause various side effects.[72] Furthermore, many patients with AD still experience recurrent relapses.[73] Therefore, new treatment approaches and drugs against new targets, without side effects, are required for the treatment of AD.

In the present study, we investigated the therapeutic effect of the HIF-1α/STAT5 decoy ODN as a new approach for AD treatment using AD *in vivo* or *in vitro* models. The synthetic HIF-1α/STAT5 decoy ODN used in this experiment was created using decoy technology. The HIF-1α/STAT5 decoy ODN was synthesized in the form of a chimeric decoy ODN, as presented in our previous study.[74,75] The chimeric decoy ODN inhibits both transcription factors by combining two different consensus sequences into one structure. When considering the introduction efficiency of ODN into mast cells, transfection of the chimeric decoy ODN may be more efficient than that of individual single decoy ODNs. As expected, we confirmed that the HIF-1α/STAT5 decoy ODN works much more effectively than the single target decoy ODN (Figures S1C and S2). On the basis of the EMSA results, the transcriptional activities of HIF-1α and STAT5 were blocked by the HIF-1α/STAT5 decoy ODN (Figures 1D–1G). The HIF-1α/STAT5 decoy ODN successfully inhibited the target genes of HIF-1α and STAT5, including VEGF, COX-2, iNOS, Bcl-2, Bcl-$_X$L, and cyclin D3, in the *in vivo* and *in vitro* AD-like skin disease models (Figures 2 and 7). In addition, we explored the therapeutic effect of the synthetic HIF-1α/STAT5 decoy ODN using a mouse model with DNCB/DfE-sensitized AD-like allergy inflammation and a IgE+Ag-treated mast-cell-like cell line.

Mast cells are long-lived tissue-resident cells that can proliferate after suitable stimuli with many inflammatory settings, including allergic reactions.[76–78] Moreover, mast cells can induce the aggregation of FcεRI (IgE/FcεRI crosslinking) and ensuing degranulation process, which is a major event in maintaining the inflammatory response.[21,79] Therefore, mast cells can be an appropriate target for the development of therapeutic agents for allergic disorders. Among the transcription factors in FcεRI signaling, HIF-1α and STAT5 play important roles in regulating mast cell survival.[34,35,56] HIF-1α is major regulator for the ability of cells to adapt to changes in oxygen level. In addition, McGettrick et al.[80] described the significance of HIF-1α in cell differentiation and survival of various immune cells under hypoxic and inflammatory conditions. STAT5 plays a crucial role in mast cell development, survival, and cytokine production, which are features of late mast cell responses. In some patients with AD, their skin lesions show accumulated mast cell infiltration with high levels of p-STAT5.[81] In addition, STAT5 is known to bind to the promoter of the Bcl-x gene and stimulate the transcription of Bcl-$_X$L, an anti-apoptosis mediator, in various immune cells.[56] Thus, we hypothesized that the HIF-1α/STAT5 decoy ODN has a suppressive effect on mast cell survival by blocking HIF-1α and STAT5 activities. In the present study, we performed TUNEL staining to detect the apoptotic cells in IgE+Ag-sensitized cells. The TUNEL staining results showed that HIF-1α/STAT5 decoy ODN treatment could effectively induce apoptosis in RBL-2H3 cells (Figure 4A). In addition, the immunoblotting results showed that the administration of the HIF-1α/STAT5 decoy ODN significantly induced the pro-apoptotic gene expression, including cytochrome *c*, cleaved-caspase-3, and Bax (Figures 4B–4E). A previous study suggested the role of STAT5a in the repression of apoptosis through the regulation of the anti-apoptotic gene Bcl-$_X$L.[82] In addition, Bcl-$_X$L is the most prominently induced gene in mouse basophils, whereas the induction of Bcl-2 is more prominent in mast cells.[83] In our study, we demonstrated that the HIF-1α/STAT5 decoy prevents the expression of the downstream factors via inhibition of the transcription activity of HIF-1α and STAT5, thus suppressing mast cell survival. In addition, we also investigated whether the HIF-1α/STAT5 decoy ODN induces apoptosis in the absence of IgE and Ag (basal state). Our results showed that the HIF-1α/STAT5 decoy ODN promote cell death in the absence of IgE+Ag (Figure 4). Furthermore, HIF-1α/STAT5 decoy ODN-induced apoptosis was enhanced in the presence of IgE+Ag compared with the absence of IgE+Ag.

Mast cells produce pro-inflammatory cytokines as allergic mediators, such as histamine, tryptase, chemokines, and cytokines.[29,84] As previously described, the activation of mast cells can result in both the degranulation and *de novo* synthesis of cytokines.[85,86] Both phases of the mast cell IgE response are key events in the pathogenesis of allergic diseases, such as AD.[87] Abraham et al.[85] reported that mast cells could produce several cytokines, including TNF-α, IL-4, IL-3, and IL-6, following appropriate stimulation. Inflammatory cytokines, including TNF-α, IL-4, IL-3, and IL-6, support a chronic phase of allergy diseases, thereby enhancing the activation of T cell or B cell survival.[29] Previous studies have suggested that the inhibition of mast cell degranulation prevents both inflammatory and allergic responses.[8,88] We hypothesized that the HIF-1α/STAT5 decoy ODN would alleviate

cells, (H) the number of degranulated mast cells, and (I) degranulated mast cell rate (degranulated/non-degranulated). The number of infiltrated or degranulated in mast cells was counted from at least 10 random fields per skin section at 400× magnification. (J) Serum IgE was measured using mouse serum (n = 5). Vehicle (Veh), distilled water; scrambled (Scr) ODN, scrambled decoy ODN; HIF-1α/STAT5 ODN, HIF-1α/STAT5 decoy ODN; NT, DNCB and DfE non-treated group; DNCB/DfE, DNCB- and DfE-sensitized group. *p < 0.05 compared with the vehicle group; †p < 0.05 compared with the DNCB/DfE-sensitized with Scr ODN group.

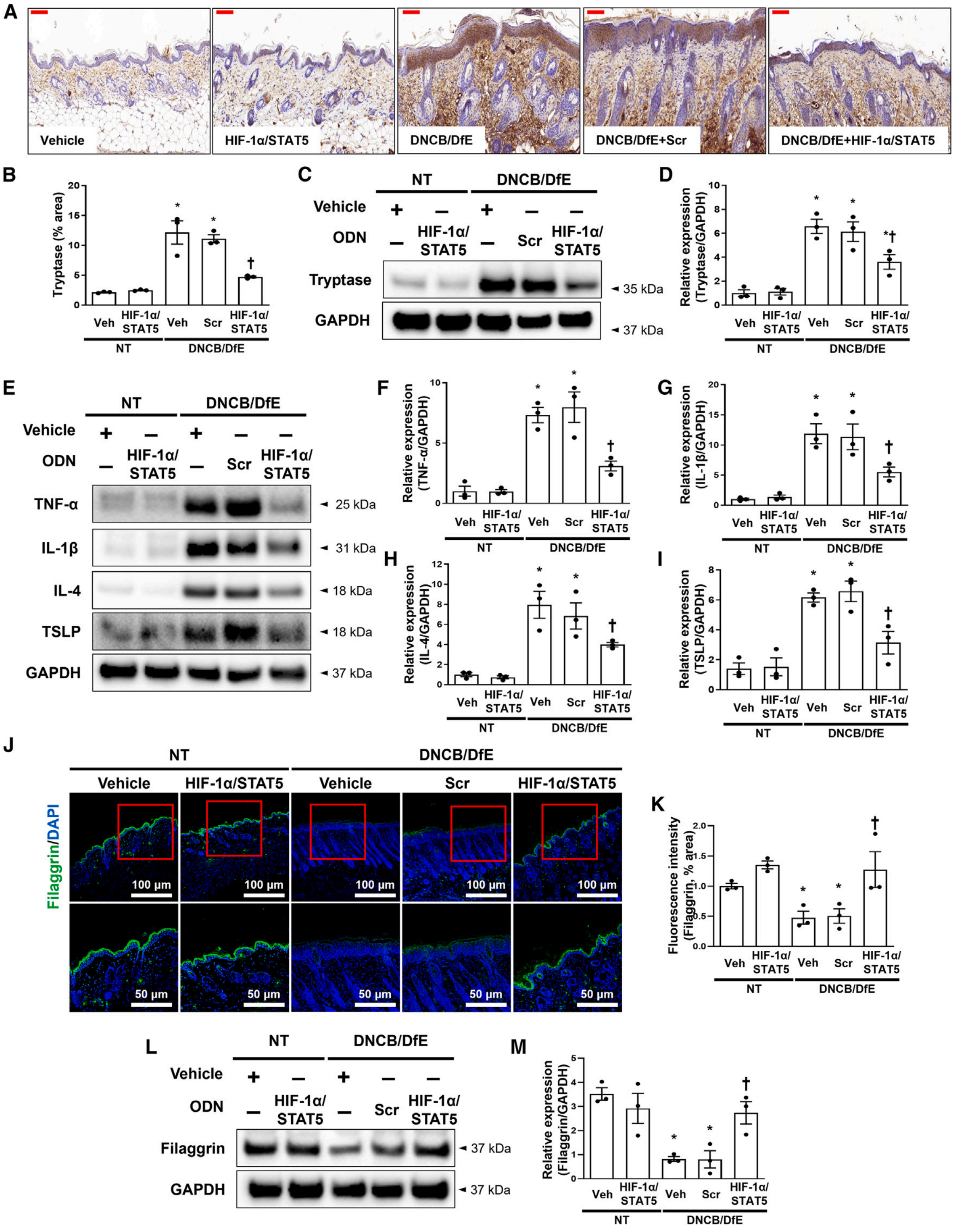

Figure 6. The HIF-1α/STAT5 decoy ODN improved the DNCB/DfE-induced Th2 inflammation and skin barrier destruction

(A) The representative tryptase in immunohistochemistry (n = 9). Scale bar, 100 μm. (B) The graphs show the tryptase-positive area (%). (C) Tryptase protein expression levels were detected using immunoblotting. GAPDH was used as a loading control. (D) The graphs show the quantification of tryptase. The dot graphs were quantified from three independent immunoblot data. (E) The protein expression levels of pro-inflammatory and Th2 cytokines were detected using immunoblotting with the indicated antibodies. GAPDH was used as a loading control. (F–I) The graphs show the quantification of (F) TNF-α, (G) IL-1β, (H) IL-4, and (I) TSLP, each normalized to GAPDH. The dot graphs were quantified from three independent immunoblot data. (J) Paraffin-embedded skin sections were deparaffinized and stained with anti-filaggrin (green) (n = 9), and the

(legend continued on next page)

AD-like symptoms by inhibiting the degranulation and cytokine synthesis of mast cells (Figures 3, 5F–5J, and 6). The immunoblotting results showed that the increased protein expression of IL-1β, IL-4, TNF-α, and tryptase in the *in vivo* and *in vitro* AD-like allergic disorder models was significantly attenuated by the HIF-1α/STAT5 decoy ODN (Figures 3 and 6). The administration of the HIF-1α/STAT5 decoy ODN effectively ameliorated mast cell infiltration and degranulation, as evidenced by Giemsa and immunohistochemistry staining with mast cell tryptase (Figures 5 and 6). The newly synthesized granules were increased by IgE/antigen stimulation, which was observed by tryptase immunofluorescence staining. However, the HIF-1α/STAT5 decoy ODN treatment suppressed the expression of tryptase, indicating the inhibition of mast cell *de novo* synthesis. Altogether, these findings suggest that the HIF-1α/STAT5 decoy ODN has an anti-inflammatory effect via the inhibition of allergic inflammatory cytokine production. However, this study has a limitation regarding the understanding the mast cell degranulation that occurred within minutes of administration, because the *in vitro* experiment was conducted at 18 h. Therefore, additional studies are required to understand the effect of the HIF-1α/STAT5 decoy ODN on mast cell degranulation.

We speculated that the HIF-1α/STAT5 decoy ODN would attenuate the AD-like skin disease by regulating the number of mast cells. To prove this hypothesis, we used a DNCB/DfE model to induce AD-like allergic inflammation. *D. farinae* is a common environmental allergen associated with AD. House dust mites are known to contribute to the pathogenesis of AD by recruiting IL-4- and IL-13-producing Th2 cells to atopic lesions.[89] Similar to other studies,[90,91] the dorsal skin of the DNCB/DfE-sensitized mice exhibited redness, hyperplasia, swelling, dysregulated differentiation of the epidermis, and infiltration of various immune cells, such as T cells and mast cells, within the lesions. The pathogenesis of AD is mediated by the interactions between the skin barrier dysfunction and abnormal inflammatory responses characterized by enhanced Th2 inflammation.[92] In addition, house dust mite allergens have been identified as a causative agent that affects skin barrier function by inducing a Th2 immune response.[60] Filaggrin is a major structural protein of the stratum corneum, which plays a crucial role in the functioning of the epidermal barrier and is influential for skin homeostasis.[93] Previous studies have shown that filaggrin deficiency and mutations affect AD and dry skin.[94] The abnormal expression of filaggrin is concerned with the impairment of the skin barrier, allowing antigens to penetrate the skin and trigger the production of inflammatory cytokines.[95–97] Our study showed that the expression of filaggrin was decreased by DNCB/DfE treatment, whereas treatment with the HIF-1α/STAT5 decoy ODN effectively ameliorated the DNCB/DfE-induced filaggrin deficiency (Figures 6J–6L). Several studies have reported that Th2 inflammatory mediators, including IL-4, IL-13, and TSLP, reduced the expression of filaggrin.[92,98] Given this finding, the HIF-1α/STAT5 decoy ODN may contribute to the prevention of epithelial barrier dysfunction by decreasing the expression levels of IL-4 and TSLP.

In summary, our results demonstrated the therapeutic effect of HIF-1α/STAT5 decoy ODN on DNCB/DfE-induced AD-like skin disease and on an IgE+Ag-sensitized mast-cell-like cell line. HIF-1α/STAT5 decoy ODN significantly suppressed HIF-1α- and STAT5-downstream genes, such as VEGF, Cox-2, iNOS, Bcl-2, Bcl-$_X$L, and cyclin D3. As a result, the HIF-1α/STAT5 decoy ODN significantly inhibited AD-like cutaneous symptoms, such as skin morphology changes, skin barrier dysfunction, and allergic inflammation. Furthermore, the HIF-1α/STAT5 decoy ODN effectively suppressed the *de novo* cytokines synthesis and induced cell death in RBL-2H3 cells (Figure 7M). To the best of our knowledge, these results are the first evidence that the simultaneous inhibition of both HIF-1α and STAT5 transcription factors using a decoy strategy effectively attenuates mast cell survival and alleviates AD-like skin disease *in vitro* and *in vivo* models. However, further studies are required to determine whether HIF-1α/STAT5 decoy ODNs may have adverse side effects, because both HIF-1α and STAT5 are essential factors for variety of cell responses. In this study, we suggest the promising possibility of HIF-1α and STAT5 as therapeutic targets and their decoy ODN as a potential therapeutic tool for AD.

MATERIALS AND METHODS

Synthesis of decoy ODNs

The decoy ODN was synthesized on a Macrogen (Seoul, Korea). The synthetic decoy ODN sequences were used as follows (the target site of the consensus sequence is underlined): scrambled (Scr) decoy ODN: 5'-GAATTCAATTCAGGGTACGGCAAAAAATTGCCGTACCCTGAATT-3'; HIF-1α decoy ODN: 5'-GAATTCGTCACGTATGAAAACATACGTGACG-3'; STAT5 decoy ODN: 5'-GAATTCTTTCCCGGAAACAAAAGTTTCCGGGAAAG-3'.

Considering the stability of the decoy ODN strategy, we designed a ring-type structured decoy ODN. These ODNs (HIF-1α, STAT5, HIF-1α/STAT5, and Scr ODN) were annealed for 6 h while temperature was gradually decreased from 80°C to 25°C. Each ODN was mixed with T4 ligase (Takara Bio, Otsu, Japan) and incubated for 18 h at 16°C to obtain a covalent ligation for the ring-type decoy ODNs.

Animals

All animal care and experimental procedures were performed and approved in accordance with the guidelines established by the

nuclei were stained with DAPI (blue). The bottom image is an enlargement of the area marked with a red square. The scale bars of the upper images are 100 μm, whereas those of the bottom images are 50 μm. (K) The immunoblotting analysis shows the expression levels of filaggrin and GAPDH in the mouse skin tissue. (L) The bar graphs show the quantitative signal intensity of filaggrin after normalization with GAPDH. The bar graphs were quantified from three independent immunoblot data. +, treated; −, untreated; vehicle (Veh), distilled water; scrambled (Scr) ODN, scrambled decoy ODN; HIF-1α/STAT5 ODN, HIF-1α/STAT5 decoy ODN; NT, DNCB and DfE non-treated group; DNCB/DfE, DNCB- and DfE-sensitized group. *$p < 0.05$ compared with the vehicle group; $^{\dagger}p < 0.05$ compared with the DNCB/DfE-sensitized group.

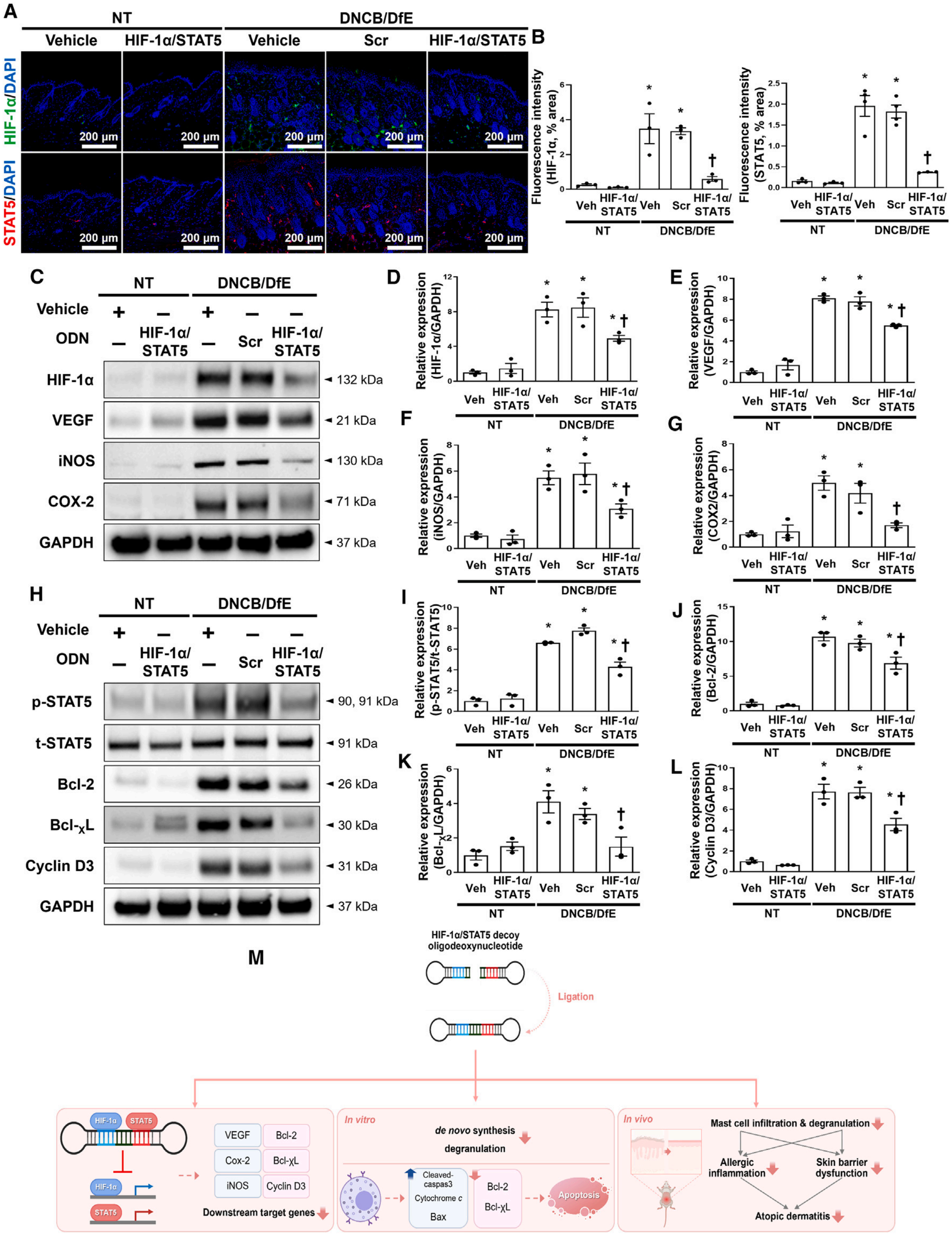

Figure 7. Inhibitory effect of the HIF-1α/STAT5 decoy ODN on HIF-1α and STAT5 expression levels and their downstream target genes in DNCB/DfE-induced AD-like skin disorder

(A) Representative images of HIF-1α (green, top) and STAT5 (red, bottom) in the mouse dorsal skin via immunofluorescence staining (n = 9). The nuclei were stained with DAPI (blue). Scale bar, 200 μm. (B) Quantification of the immunofluorescence signals of HIF-1α and STAT5. (C) Western blot analysis of HIF-1α and HIF-1α-related genes, including VEGF, iNOS, and COX-2. GAPDH was used as a loading control. (D–G) The dot graphs show the quantitative signal intensity of (D) HIF-1α, (E) VEGF, (F) iNOS, and (G) COX-2

(legend continued on next page)

Institutional Animal Care and Use Committee of the Catholic University of Daegu (Approval number DCIAFCR-190620-08-Y). The animals were treated humanely, and every effort was made to minimize the animals' suffering and the number of animals used. Six-week-old female Balb/c mice (Samtoko, Osan, Korea) were housed in polycarbonate animal cages and maintained under conventional conditions at 22 ± 2°C and 55% humidity and allowed to acclimatize for one week. The mice were randomly divided into each group (n = 9 per group).

Induction of AD animal model and synthetic decoy ODN administration

The Balb/c mice were anesthetized by isoflurane inhalation (Ifran; HANA Pharm, Seoul, Korea) using an RC2 Rodent Cir-cuit Controller (VETEQUIP). The dorsal skin was shaved under anesthesia using an electric clipper and hair removal creams. On the first day after shaving, 200 μL 1% DNCB in acetone/olive oil (3:1) was applied to the shaved dorsal skin. The following week, 200 μL 1% DNCB was applied to the dorsal skin, followed four days later by 200 μL DfE (10 mg/mL). DNCB/DfE treatment was repeated weekly in rotation for four weeks. One week after the first induction of AD, HIF-1α, STAT5, HIF-1α/STAT5, and Scr ODN (10 μg) were injected into the mice intravenously at an interval of once time per week for four weeks using an *in vivo* gene delivery system (Mirus Bio, Madison, WI). The dose of synthetic ODN used in this study were based on previous studies.[47] The experimental procedure for the induction of AD-like skin inflammation is schematically described in Figure 5A. The mice were sacrificed at the end of each treatment period. Blood samples were obtained by puncturing the heart. Immediately following blood collection, the dorsal skin was excised for the subsequent experiments.

Transfection of synthetic decoy ODNs and transfection efficiency

To evaluate the *in vivo* transcription efficiency of the synthetic decoy ODNs, mice were injected with FITC-labeled decoy ODN via the tail vein. Before transfection, the HIF-1α/STAT5 decoy ODN was labeled with FITC using a Label IT nucleic acid-labeling kit (Mirus Bio). Skin samples were embedded using an optimum cutting temperature compound (Sakura Finetek Japan, Tokyo, Japan), frozen sectioning was performed. Fluorescence was measured using a confocal microscope (Nikon, Tokyo, Japan). The *in vitro* transfection efficiency of the HIF-1α/STAT5 decoy ODN was examined using confocal microscopy and flow cytometry (Navious; Beckman Coulter, Miami, FL). For fluorescence analysis, RBL-2H3 cells were cultured in six-well plates (2×10^5 cells/well) and transfected with FITC-labeled ODN using Lipofectamine 2000 (Invitrogen, Carlsbad, CA). After transfection for 6 h, the cells were washed with PBS and fixed with 3.75% paraformaldehyde for 15 min at room temperature (RT). To investigate the persistence of the synthetic decoy ODN, the RBL-2H3 cells were washed and incubated with serum-free medium for 24 h after 6 h of transfection. The fixed cells were stained with DAPI for 2 min, and the slides were mounted using Dako fluorescence mounting medium. Specimens were examined and photographed with a Nikon Alt confocal microscope (Nikon). For flow cytometric analysis, RBL-2H3 cells were seeded at 3×10^5 cells per 3 mL of complete medium in a 60 mm dish and transfected with FITC-labeled ODNs. After transfection for 6 h, the cells were washed, trypsinized, dispersed, and then transferred into 500 μL PBS. The samples were then analyzed using flow cytometry.

Cell culture and treatments

The RBL-2H3 cell line was obtained from the American Type Culture Collection (ATCC). The RBL-2H3 cells were cultured in DMEM supplemented with 10% fetal bovine serum (FBS) and 1% antibiotics (100 U/mL penicillin and 100 μg/mL streptomycin) at 37°C in a 5% humidified CO_2 incubator. The RBL-2H3 cells were seeded at 3×10^5 cells in a 60 mm cell culture dish and sensitized with anti-DNP-IgE (100 ng/mL). After incubating overnight, the medium was changed to serum-free medium containing the indicated synthetic decoy ODNs (60 nM) with Lipofectamine 2000. After transfection for 6 h, the RBL-2H3 cells were cultured in a serum-free medium containing DNP-BSA (10 μg/mL) for an additional 18 h.

ELISA

The whole-blood samples collected via cardiac puncture were allowed to clot for 1 h at RT. Sera for the immunoassays were obtained from the supernatants after centrifugation at 2,000 × *g* for 20 min. Next, using an ELISA kit in accordance with the manufacturer's instructions; the IgE concentration in mouse serum was measured. Optical density was measured at 450 nm using an ELISA reader (BMG Labtech, Baden-Wurttemberg, Germany). Measurements were performed in triplicate and the IgE concentrations of the samples were determined by comparison with the standard curve. The ELISA kits were purchased from Bethyl Laboratories (IgE; Montgomery, TX, USA).

Histological analysis

All dorsal skin tissues were fixed in 10% formalin solution for 24 h at RT. After fixation, the sections that were cut perpendicular to the anterior-posterior axis of the skin were dehydrated in graded ethanol, cleared in xylene, and embedded in paraffin. Subsequently, paraffin-embedded skin tissues were cut into 4 μm sections, mounted on slides, and deparaffinized. The sections were stained with H&E to

after normalization with GAPDH. The graphs were quantified from three immunoblot data. (H) The protein expression levels of STAT5 and STAT5-targeted genes, including Bcl2, Bcl-$_X$L, and cyclin D3, were analyzed using immunoblotting. GAPDH was used as a loading control. (I–L) The bar graphs show the quantitative signal intensity of (I) STAT5, (J) Bcl2, (K) Bcl-$_X$L, and (L) cyclin D3 after normalization with GAPDH or t-STAT5. The dot graphs were quantified from three independent immunoblot data. (M) The graphic illustration of the effect of HIF-1α/STAT5 decoy ODN in DNCB/DfE-induced and IgE/Ag-induced AD-like model via suppressing of transcriptional expression. +, treated; −, un-treated; vehicle (Veh), distilled water; scrambled (Scr) ODN, scrambled decoy ODN; HIF-1α/STAT5 ODN, HIF-1α/STAT5 decoy ODN; NT, DNCB and DfE non-treated group; DNCB/DfE, DNCB- and DfE-sensitized group. *$p < 0.05$ compared with the vehicle group; $^{\dagger}p < 0.05$ compared with the DNCB/DfE-sensitized group.

analyze the thicknesses of the epidermis, dermis, and inflammatory infiltration; they were also stained with Giemsa to count the mast cells. As part of the histological assessment, all slides were examined under a slide scanner (3DHISTECH Pannoramic MIDI, Budapest, Hungary). The thicknesses of the epidermis and dermis were measured from at least 10 random fields per section at 200× magnification with CaseViewer 1.4 software (3DHISTECH). The number of infiltrated and degranulated mast cells was counted from at least 10 random fields per section at 400× magnification with CaseViewer 1.4 software.

Immunohistochemical staining

The paraffin-embedded tissue sections on the slides were deparaffinized with xylene and dehydrated with gradually decreasing concentrations of ethanol. The dehydrated tissue sections were treated with 3% hydrogen peroxide in methanol for 10 min to block endogenous peroxidase activity. The tissue sections were immersed in 10 mM sodium citrate buffer (pH 6.0) for 5 min at 95°C. The final step was repeated using a 10 mM sodium citrate solution (pH 6.0). The sections stayed in the same solution while cooling for 20 min and then rinsed with PBS. Subsequently, the sections were incubated with a primary antibody (1:100 dilution) for 1 h at 37°C. The primary antibodies were used anti-TNF-α and anti-tryptase (Abcam, Cambridge, MA). After three serial washes with PBS, the signal was visualized using an Envision System (DAKO) for 30 min at 37°C. 3,3′-Diaminobenzidine tetrahydrochloride was used as a coloring reagent, and hematoxylin was used as the counterstain. The slides were viewed with a slide scanner (Pannoramic MIDI) and the integrated optical density was analyzed using the iSolution DT software.

Immunofluorescence staining and confocal microscopy

The paraffin-embedded skin tissue sections were placed on slides and deparaffinized. The skin tissue sections were placed in a blocking solution (1% BSA in PBS) at RT for 30 min. The slides were immunostained with the primary antibodies (1:200) against Filaggrin (Enzo Life Sciences), HIF-1α (Santa Cruz Biotechnology, Santa Cruz, CA), or p-STAT5 (Santa Cruz Biotechnology) as appropriate at 37°C for 1 h. After washing, the sections were incubated with secondary antibodies (1:200 dilution) conjugated with Alexa Fluor 488/Alexa Fluor 555 (Thermo Fisher Scientific, Waltham, MA) for 1 h at 37°C. The nuclei were labeled with DAPI (1:1,000) at RT for 2 min and the tissue slides were mounted using a Dako fluorescence mounting medium. The stained slides were viewed under a confocal microscope (Nikon). The treated RBL-2H3 cells were washed with PBS and fixed with 3.75% paraformaldehyde for 15 min at RT. The fixed cells were then permeabilized by treating them with 0.1% Triton X-100 in PBS for 10 min. Following permeabilization, the cells were blocked in PBS containing 1% BSA at RT for 15 min. After blocking, the cells were incubated with a primary antibody (1:200 dilution) against HIF-1α (Santa Cruz Biotechnology), tryptase (Abcam), or p-STAT5 (Santa Cruz Biotechnology) at 37°C for 1 h. The cells were incubated with a secondary antibody (Alexa Fluor 488 or Alexa Fluor 555) at 37°C for 1 h and the nuclei were stained with DAPI for 2 min. The slides were mounted using Dako fluorescence mounting medium, and the specimens were examined and photographed using a confocal microscope system (Nikon). To quantify all immunofluorescence results, we measured at least 3 areas of the immunofluorescence image.

TUNEL staining

Apoptotic cell death was verified by TUNEL staining using an *in situ* cell death detection kit (Roche Diagnostics, Indianapolis, IN) according to the manufacturer's instructions. The RBL-2H3 cells were seeded at 2×10^5 cells per 2 mL in a six-well cell culture dish. After transfection and stimulation, the cells were washed with PBS and fixed with 3.75% paraformaldehyde for 15 min at RT. The fixed cells were permeabilized with 0.1% Triton X-100 in 0.1% sodium citrate for 10 min at RT. After washing, the cells were incubated with the TUNEL reaction mixture for 1 h at 37°C. Nuclei were stained with DAPI and images were visualized and captured using a confocal microscope (Nikon).

EMSA

The nuclear protein fractionation from the RBL-2H3 cells and skin tissues of mice was conducted using an NE-PER Nuclear and Cytoplasmic Extraction Kit (Thermo Fisher Scientific) according to the instruction manual. The Lightshift Chemiluminescent EMSA Kit (Thermo Fisher Scientific) was used for the EMSA assay to analyze the DNA-binding activity of HIF-1α and STAT5. The HIF-1α and STAT5 (HIF-1α forward: 5'-TCTGT<u>ACGTG</u>ACCACACTCACCTC-3'; HIF-1α reverse: 5'-GAGGTGAGTGTGGT<u>CACGT</u>ACAGA-3'; STAT5 forward: 5'-TCTCT<u>TTCCCGGAA</u>ACTC-3'; STAT5 reverse: 5'-GAGT<u>TTCCGGGAA</u>AGAGA-3'; consensus sequences are underlined) oligonucleotide probe was 3'-end-labeled with biotin. An image analyzer (Chemidoc XRS+system) was used to detect the chemiluminescence of the biotin-labeled DNA.

Immunoblot analysis

Protein samples were prepared from the skin tissues and cultured RBL-2H3 cells using a protein extraction buffer (Cell Lytic M; Sigma-Aldrich) according to the manufacturer's instructions. The total protein concentration of the samples at an optimal density of 562 nm, as measured using a spectrophotometer, was measured using a BCA Protein Assay (Thermo Fisher Scientific). The protein samples were separated on a precast gradient polyacrylamide gel (Bolt 4%–12% Bis-Tris Plus Gels, Thermo Fisher Scientific) and transferred to a nitrocellulose membrane (GenDEPOT, Barker, TX) using a Bolt Mini Blot Module, Mini Gel Tank, and Power Blotter-Semi-dry Transfer System (Thermo Fisher Scientific) according to the manufacturer's recommendations. The membranes were blocked with 5% BSA in Tris-buffered saline with Tween 20 (TBS-T; 10 mM Tris, 150 mM NaCl, and 0.1% Tween 20) for 1 h at RT. The blocked membranes were incubated with a primary antibody (1:1,000 dilution) overnight at 4°C. The membranes were washed and probed with horseradish peroxidase (HRP)-conjugated secondary antibody (1:1,000 dilution) for 1 h at RT. Following another wash step, the membranes were maintained in an enhanced chemiluminescence detection reagent (Thermo Fisher Scientific). The signals were detected using an enhanced chemiluminescence detection system

(Amersham, Piscataway, NJ). The protein expression values were normalized to the GAPDH expression values. The primary antibodies used in this study were as follows: anti-Bax, Bcl-2, COX-2, HIF-1α, IL-4, IL-1β, iNOS, VEGF (Santa Cruz Biotechnology), anti-TNF-α, tryptase, TSLP, p-STAT5, t-STAT5 (Abcam), anti-Bcl-$_X$L, cleaved caspase-3, cyclin D3, cytochrome *c*, GAPDH (Cell Signaling, Beverly, MA), and anti-filaggrin (Enzo Life Sciences).

Statistical analysis

All data are presented as mean ± SEM. Group means were compared using one-way analysis of variance (ANOVA) with Tukey's multiple-comparison test. Statistical significance was examined using Prism 5 (GraphPad Software, Inc., San Diego, CA). A p value of <0.05 was determined to indicate statistical significance.

DATA AND CODE AVAILABILITY

The authors confirm that the data supporting the findings of this study are available within the article and its supplemental material.

SUPPLEMENTAL INFORMATION

Supplemental information can be found online at https://doi.org/10.1016/j.omtn.2023.102036.

ACKNOWLEDGMENTS

This work was supported by a National Research Foundation of Korea grant funded by the Korean Government (NRF-2019R1F1A1061019 and NRF-2022R1C1C2010960).

AUTHOR CONTRIBUTIONS

K.-K.P., J.L., and M.-G.G. participated in the study conception and design. M.-G.G., J.L., and J.H.K. analyzed data and results. H.-J.A., H.G., S.B., and M.-G.G. performed animal and cell experiments. M.-G.G. and J.H.K. contributed to the original draft. K.-K.P., M.-G.G., and J.H.K. edited the manuscript. All authors read and approved the final version of manuscript.

DECLARATION OF INTERESTS

The authors declare no competing interests.

REFERENCES

1. Asher, M.I., Montefort, S., Björkstén, B., Lai, C.K.W., Strachan, D.P., Weiland, S.K., and Williams, H.; ISAAC Phase Three Study Group (2006). Worldwide time trends in the prevalence of symptoms of asthma, allergic rhinoconjunctivitis, and eczema in childhood: ISAAC Phases One and Three repeat multicountry cross-sectional surveys. Lancet *368*, 733–743.
2. Lim, J.Y., Lee, J.H., Lee, D.H., Lee, J.H., and Kim, D.K. (2019). Umbelliferone reduces the expression of inflammatory chemokines in HaCaT cells and DNCB/DFE-induced atopic dermatitis symptoms in mice. Int. Immunopharm. *75*, 105830.
3. Sehra, S., Serezani, A.P.M., Ocaña, J.A., Travers, J.B., and Kaplan, M.H. (2016). Mast Cells Regulate Epidermal Barrier Function and the Development of Allergic Skin Inflammation. J. Invest. Dermatol. *136*, 1429–1437.
4. Flohr, C., and Mann, J. (2014). New insights into the epidemiology of childhood atopic dermatitis. Allergy *69*, 3–16.
5. Drube, S., Grimlowski, R., Deppermann, C., Fröbel, J., Kraft, F., Andreas, N., Stegner, D., Dudeck, J., Weber, F., Rödiger, M., et al. (2017). The Neurobeachin-like 2 Protein Regulates Mast Cell Homeostasis. J. Immunol. *199*, 2948–2957.
6. Leung, D.Y.M., and Bieber, T. (2003). Atopic dermatitis. Lancet *361*, 151–160.
7. Yamamoto, M., Haruna, T., Yasui, K., Takahashi, H., Iduhara, M., Takaki, S., Deguchi, M., and Arimura, A. (2007). A novel atopic dermatitis model induced by topical application with dermatophagoides farinae extract in NC/Nga mice. Allergol. Int. *56*, 139–148.
8. Kawakami, T., and Galli, S.J. (2002). Regulation of mast-cell and basophil function and survival by IgE. Nat. Rev. Immunol. *2*, 773–786.
9. Valenta, R., Natter, S., Seiberler, S., Wichlas, S., Maurer, D., Hess, M., Pavelka, M., Grote, M., Ferreira, F., Szepfalusi, Z., et al. (1998). Molecular characterization of an autoallergen, Hom s 1, identified by serum IgE from atopic dermatitis patients. J. Invest. Dermatol. *111*, 1178–1183.
10. Choi, J.H., Jin, S.W., Han, E.H., Park, B.H., Kim, H.G., Khanal, T., Hwang, Y.P., Do, M.T., Lee, H.S., Chung, Y.C., et al. (2014). Platycodon grandiflorum root-derived saponins attenuate atopic dermatitis-like skin lesions via suppression of NF-kappaB and STAT1 and activation of Nrf2/ARE-mediated heme oxygenase-1. Phytomedicine *21*, 1053–1061.
11. Choi, J.K., Oh, H.M., Lee, S., Kwon, T.K., Shin, T.Y., Rho, M.C., and Kim, S.H. (2014). Salvia plebeia suppresses atopic dermatitis-like skin lesions. Am. J. Chin. Med. *42*, 967–985.
12. Lee, H.J., Lee, N.R., Jung, M., Kim, D.H., and Choi, E.H. (2015). Atopic March from Atopic Dermatitis to Asthma-Like Lesions in NC/Nga Mice Is Accelerated or Aggravated by Neutralization of Stratum Corneum but Partially Inhibited by Acidification. J. Invest. Dermatol. *135*, 3025–3033.
13. Guttman-Yassky, E., and Krueger, J.G. (2017). Atopic dermatitis and psoriasis: two different immune diseases or one spectrum? Curr. Opin. Immunol. *48*, 68–73.
14. Brandt, E.B., and Sivaprasad, U. (2011). Th2 Cytokines and Atopic Dermatitis. J. Clin. Cell. Immunol. *2*, 110.
15. Walczak-Drzewiecka, A., Ratajewski, M., Wagner, W., and Dastych, J. (2008). HIF-1alpha is up-regulated in activated mast cells by a process that involves calcineurin and NFAT. J. Immunol. *181*, 1665–1672.
16. Mekori, Y.A., and Metcalfe, D.D. (2000). Mast cells in innate immunity. Immunol. Rev. *173*, 131–140.
17. Theoharides, T.C., and Kalogeromitros, D. (2006). The critical role of mast cells in allergy and inflammation. Ann. N. Y. Acad. Sci. *1088*, 78–99.
18. Amin, K. (2012). The role of mast cells in allergic inflammation. Respir. Med. *106*, 9–14.
19. Crivellato, E., Travan, L., and Ribatti, D. (2010). Mast cells and basophils: a potential link in promoting angiogenesis during allergic inflammation. Int. Arch. Allergy Immunol. *151*, 89–97.
20. Furue, M., Chiba, T., Tsuji, G., Ulzii, D., Kido-Nakahara, M., Nakahara, T., and Kadono, T. (2017). Atopic dermatitis: immune deviation, barrier dysfunction, IgE autoreactivity and new therapies. Allergol. Int. *66*, 398–403.
21. Kobayasi, T., and Asboe-Hansen, G. (1969). Degranulation and regranulation of human mast cells. An electron microscopic study of the whealing reaction in urticaria pigmentosa. Acta Derm. Venereol. *49*, 369–381.
22. Kurosawa, M., Inamura, H., Kanbe, N., Igarashi, Y., Tomita, T., Takeda, J., and Miyachi, Y. (1998). Phase-contrast microscopic studies using cinematographic techniques and scanning electron microscopy on IgE-mediated degranulation of cultured human mast cells. Clin. Exp. Allergy *28*, 1007–1012.
23. Ekoff, M., Lyberg, K., Krajewska, M., Arvidsson, M., Rak, S., Reed, J.C., Harvima, I., and Nilsson, G. (2012). Anti-apoptotic BFL-1 is the major effector in activation-induced human mast cell survival. PLoS One *7*, e39117.
24. Hazzan, T., Eberle, J., Worm, M., and Babina, M. (2019). Thymic Stromal Lymphopoietin Interferes with the Apoptosis of Human Skin Mast Cells by a Dual Strategy Involving STAT5/Mcl-1 and JNK/Bcl-xL. Cells *8*.
25. Ekoff, M., and Nilsson, G. (2011). Mast cell apoptosis and survival. Adv. Exp. Med. Biol. *716*, 47–60.
26. Pullen, N.A., Barnstein, B.O., Falanga, Y.T., Wang, Z., Suzuki, R., Tamang, T.D.L., Khurana, M.C., Harry, E.A., Draber, P., Bunting, K.D., et al. (2012). Novel mechanism for Fc{epsilon}RI-mediated signal transducer and activator of transcription 5 (STAT5) tyrosine phosphorylation and the selective influence of STAT5B over mast cell cytokine production. J. Biol. Chem. *287*, 2045–2054.

27. Kinet, J.P. (1999). The high-affinity IgE receptor (Fc epsilon RI): from physiology to pathology. Annu. Rev. Immunol. *17*, 931–972.

28. Kitaura, J., Eto, K., Kinoshita, T., Kawakami, Y., Leitges, M., Lowell, C.A., and Kawakami, T. (2005). Regulation of highly cytokinergic IgE-induced mast cell adhesion by Src, Syk, Tec, and protein kinase C family kinases. J. Immunol. *174*, 4495–4504.

29. Kalesnikoff, J., and Galli, S.J. (2008). New developments in mast cell biology. Nat. Immunol. *9*, 1215–1223.

30. Choi, Y.H., Jin, G.Y., Li, L.C., and Yan, G.H. (2013). Inhibition of protein kinase C delta attenuates allergic airway inflammation through suppression of PI3K/Akt/mTOR/HIF-1 alpha/VEGF pathway. PLoS One *8*, e81773.

31. Ivan, M., Kondo, K., Yang, H., Kim, W., Valiando, J., Ohh, M., Salic, A., Asara, J.M., Lane, W.S., and Kaelin, W.G., Jr. (2001). HIFalpha targeted for VHL-mediated destruction by proline hydroxylation: implications for O2 sensing. Science *292*, 464–468.

32. Kim, H.R., Kim, J.H., Choi, E.J., Lee, Y.K., Kie, J.H., Jang, M.H., and Seoh, J.Y. (2014). Hyperoxygenation attenuated a murine model of atopic dermatitis through raising skin level of ROS. PLoS One *9*, e109297.

33. Zhou, H., Chen, X., Zhang, W.M., Zhu, L.P., and Cheng, L. (2012). HIF-1alpha inhibition reduces nasal inflammation in a murine allergic rhinitis model. PLoS One *7*, e48618.

34. Semenza, G.L. (2003). Targeting HIF-1 for cancer therapy. Nat. Rev. Cancer *3*, 721–732.

35. Egger, M., Schgoer, W., Beer, A.G.E., Jeschke, J., Leierer, J., Theurl, M., Frauscher, S., Tepper, O.M., Niederwanger, A., Ritsch, A., et al. (2007). Hypoxia up-regulates the angiogenic cytokine secretoneurin via an HIF-1alpha- and basic FGF-dependent pathway in muscle cells. Faseb. J. *21*, 2906–2917.

36. Sumbayev, V.V., Nicholas, S.A., Streatfield, C.L., and Gibbs, B.F. (2009). Involvement of hypoxia-inducible factor-1 HiF(1alpha) in IgE-mediated primary human basophil responses. Eur. J. Immunol. *39*, 3511–3519.

37. Yuan, G., Nanduri, J., Khan, S., Semenza, G.L., and Prabhakar, N.R. (2008). Induction of HIF-1alpha expression by intermittent hypoxia: involvement of NADPH oxidase, Ca2+ signaling, prolyl hydroxylases, and mTOR. J. Cell. Physiol. *217*, 674–685.

38. Huang, I.H., Chung, W.H., Wu, P.C., and Chen, C.B. (2022). JAK-STAT signaling pathway in the pathogenesis of atopic dermatitis: An updated review. Front. Immunol. *13*, 1068260.

39. Spinelli, F.R., Colbert, R.A., and Gadina, M. (2021). JAK1: Number one in the family; number one in inflammation? Rheumatology *60*, ii3–ii10.

40. Dubin, C., Del Duca, E., and Guttman-Yassky, E. (2021). The IL-4, IL-13 and IL-31 pathways in atopic dermatitis. Expet Rev. Clin. Immunol. *17*, 835–852.

41. Banik, S., Rakshit, S., and Sarkar, K. (2021). The Role of STAT1 in T Helper Cell Differentiation during Breast Cancer Progression. J. Breast Cancer *24*, 253–265.

42. Shelburne, C.P., McCoy, M.E., Piekorz, R., Sexl, V., Roh, K.H., Jacobs-Helber, S.M., Gillespie, S.R., Bailey, D.P., Mirmonsef, P., Mann, M.N., et al. (2003). Stat5 expression is critical for mast cell development and survival. Blood *102*, 1290–1297.

43. Darnell, J.E., Jr. (1997). STATs and gene regulation. Science *277*, 1630–1635.

44. Morishita, R., Sugimoto, T., Aoki, M., Kida, I., Tomita, N., Moriguchi, A., Maeda, K., Sawa, Y., Kaneda, Y., Higaki, J., and Ogihara, T. (1997). In vivo transfection of cis element "decoy" against nuclear factor-kappaB binding site prevents myocardial infarction. Nat. Med. *3*, 894–899.

45. An, H.J., Tizaoui, K., Terrazzino, S., Cargnin, S., Lee, K.H., Nam, S.W., Kim, J.S., Yang, J.W., Lee, J.Y., Smith, L., et al. (2020). Beneficial Effects of SREBP Decoy Oligodeoxynucleotide in an Animal Model of Hyperlipidemia. Int. J. Mol. Sci. *21*, 5678.

46. Mann, M.J., and Dzau, V.J. (2000). Therapeutic applications of transcription factor decoy oligonucleotides. J. Clin. Invest. *106*, 1071–1075.

47. Kim, J.Y., An, H.J., Kim, W.H., Gwon, M.G., Gu, H., Park, Y.Y., and Park, K.K. (2017). Anti-fibrotic Effects of Synthetic Oligodeoxynucleotide for TGF-beta1 and Smad in an Animal Model of Liver Cirrhosis. Mol. Ther. Nucleic Acids *8*, 250–263.

48. Cao, C.C., Ding, X.Q., Ou, Z.L., Liu, C.F., Li, P., Wang, L., and Zhu, C.F. (2004). In vivo transfection of NF-kappaB decoy oligodeoxynucleotides attenuate renal ischemia/reperfusion injury in rats. Kidney Int. *65*, 834–845.

49. Miyake, T., Aoki, M., Nakashima, H., Kawasaki, T., Oishi, M., Kataoka, K., Tanemoto, K., Ogihara, T., Kaneda, Y., and Morishita, R. (2006). Prevention of abdominal aortic aneurysms by simultaneous inhibition of NFkappaB and ets using chimeric decoy oligonucleotides in a rabbit model. Gene Ther. *13*, 695–704.

50. Gwon, M.G., An, H.J., Kim, J.Y., Kim, W.H., Gu, H., Kim, H.J., Leem, J., Jung, H.J., and Park, K.K. (2020). Anti-fibrotic effects of synthetic TGF-beta1 and Smad oligodeoxynucleotide on kidney fibrosis *in vivo* and *in vitro* through inhibition of both epithelial dedifferentiation and endothelial-mesenchymal transitions. Faseb. J. *34*, 333–349.

51. Yagil, Z., Kay, G., Nechushtan, H., and Razin, E. (2009). A specific epitope of protein inhibitor of activated STAT3 is responsible for the induction of apoptosis in rat transformed mast cells. J. Immunol. *182*, 2168–2175.

52. Jung, Y.J., Isaacs, J.S., Lee, S., Trepel, J., and Neckers, L. (2003). IL-1beta-mediated up-regulation of HIF-1alpha via an NFkappaB/COX-2 pathway identifies HIF-1 as a critical link between inflammation and oncogenesis. Faseb. J. *17*, 2115–2117.

53. Gibbs, B.F., Yasinska, I.M., Oniku, A.E., and Sumbayev, V.V. (2011). Effects of stem cell factor on hypoxia-inducible factor 1 alpha accumulation in human acute myeloid leukaemia and LAD2 mast cells. PLoS One *6*, e22502.

54. Bischoff, S.C. (2007). Role of mast cells in allergic and non-allergic immune responses: comparison of human and murine data. Nat. Rev. Immunol. *7*, 93–104.

55. Guttman-Yassky, E., Nograles, K.E., and Krueger, J.G. (2011). Contrasting pathogenesis of atopic dermatitis and psoriasis–part I: clinical and pathologic concepts. J. Allergy Clin. Immunol. *127*, 1110–1118.

56. Morales, J.K., Falanga, Y.T., Depcrynski, A., Fernando, J., and Ryan, J.J. (2010). Mast cell homeostasis and the JAK-STAT pathway. Gene Immun. *11*, 599–608.

57. Harris, A.J., Thompson, A.R., Whyte, M.K., and Walmsley, S.R. (2014). HIF-mediated innate immune responses: cell signaling and therapeutic implications. Hypoxia *2*, 47–58.

58. Sumbayev, V.V., Nicholas, S.A., and Gibbs, B.F. (2010). Differential role of hypoxia-inducible factor 1 alpha in toll-like receptor-mediated and allergic inflammatory reactions. World Allergy Organ. J. *3*, 245–249.

59. Dai, X., Sayama, K., Tohyama, M., Shirakata, Y., Hanakawa, Y., Tokumaru, S., Yang, L., Hirakawa, S., and Hashimoto, K. (2011). Mite allergen is a danger signal for the skin via activation of inflammasome in keratinocytes. J. Allergy Clin. Immunol. *127*, 806–814.e1-4-804.

60. Choi, J.K., and Kim, S.H. (2013). Rutin suppresses atopic dermatitis and allergic contact dermatitis. Exp. Biol. Med. *238*, 410–417.

61. Kwon, H.K., Lee, C.G., So, J.S., Chae, C.S., Hwang, J.S., Sahoo, A., Nam, J.H., Rhee, J.H., Hwang, K.C., and Im, S.H. (2010). Generation of regulatory dendritic cells and CD4+Foxp3+ T cells by probiotics administration suppresses immune disorders. Proc. Natl. Acad. Sci. USA *107*, 2159–2164.

62. Liu, X., Wang, J., Zhang, H., Zhan, M., Chen, H., Fang, Z., Xu, C., Chen, H., and He, S. (2016). Induction of Mast Cell Accumulation by Tryptase via a Protease Activated Receptor-2 and ICAM-1 Dependent Mechanism. Mediat. Inflamm. *2016*, 6431574.

63. McLeod, D.S., Bhutto, I., Edwards, M.M., Gedam, M., Baldeosingh, R., and Lutty, G.A. (2017). Mast Cell-Derived Tryptase in Geographic Atrophy. Invest. Ophthalmol. Vis. Sci. *58*, 5887–5896.

64. Jin, W., Huang, W., Chen, L., Jin, M., Wang, Q., Gao, Z., and Jin, Z. (2018). Topical Application of JAK1/JAK2 Inhibitor Momelotinib Exhibits Significant Anti-Inflammatory Responses in DNCB-Induced Atopic Dermatitis Model Mice. Int. J. Mol. Sci. *19*, 3973.

65. Elias, P.M., and Schmuth, M. (2009). Abnormal skin barrier in the etiopathogenesis of atopic dermatitis. Curr. Allergy Asthma Rep. *9*, 265–272.

66. Flohr, C., and Yeo, L. (2011). Atopic dermatitis and the hygiene hypothesis revisited. Curr. Probl. Dermatol. *41*, 1–34.

67. Takizawa, H. (2011). Impact of air pollution on allergic diseases. Korean J. Intern. Med. (Engl. Ed.) *26*, 262–273.

68. Larsen, J.N., Broge, L., and Jacobi, H. (2016). Allergy immunotherapy: the future of allergy treatment. Drug Discov. Today *21*, 26–37.

69. Dhami, S., Kakourou, A., Asamoah, F., Agache, I., Lau, S., Jutel, M., Muraro, A., Roberts, G., Akdis, C.A., Bonini, M., et al. (2017). Allergen immunotherapy for allergic asthma: A systematic review and meta-analysis. Allergy *72*, 1825–1848.

70. Jousilahti, P., Haahtela, T., Laatikainen, T., Mäkelä, M., and Vartiainen, E. (2016). Asthma and respiratory allergy prevalence is still increasing among Finnish young adults. Eur. Respir. J. *47*, 985–987.

71. Yu, S.H., Drucker, A.M., Lebwohl, M., and Silverberg, J.I. (2018). A systematic review of the safety and efficacy of systemic corticosteroids in atopic dermatitis. J. Am. Acad. Dermatol. *78*, 733–740.e11.

72. Kang, J., Lee, S., Kim, N., Dhakal, H., Choi, Y.A., Kwon, T.K., Khang, D., and Kim, S.H. (2021). Hispidulin alleviates 2,4-dinitrochlorobenzene and house dust mite extract-induced atopic dermatitis-like skin inflammation. Biomed. Pharmacother. *137*, 111359.

73. Del Rosso, J., and Friedlander, S.F. (2005). Corticosteroids: options in the era of steroid-sparing therapy. J. Am. Acad. Dermatol. *53*, S50–S58.

74. Kim, K.H., Park, J.H., Lee, W.R., Park, J.S., Kim, H.C., and Park, K.K. (2013). The inhibitory effect of chimeric decoy oligodeoxynucleotide against NF-kappaB and Sp1 in renal interstitial fibrosis. J. Mol. Med. *91*, 573–586.

75. Sung, W.J., Kim, K.H., Kim, Y.J., Chang, Y.C., Lee, I.H., and Park, K.K. (2013). Antifibrotic effect of synthetic Smad/Sp1 chimeric decoy oligodeoxynucleotide through the regulation of epithelial mesenchymal transition in unilateral ureteral obstruction model of mice. Exp. Mol. Pathol. *95*, 136–143.

76. Kitamura, Y. (1989). Heterogeneity of mast cells and phenotypic change between subpopulations. Annu. Rev. Immunol. *7*, 59–76.

77. Galli, S.J., Kalesnikoff, J., Grimbaldeston, M.A., Piliponsky, A.M., Williams, C.M.M., and Tsai, M. (2005). Mast cells as "tunable" effector and immunoregulatory cells: recent advances. Annu. Rev. Immunol. *23*, 749–786.

78. Ryan, J.J., Kashyap, M., Bailey, D., Kennedy, S., Speiran, K., Brenzovich, J., Barnstein, B., Oskeritzian, C., and Gomez, G. (2007). Mast cell homeostasis: a fundamental aspect of allergic disease. Crit. Rev. Immunol. *27*, 15–32.

79. Dvorak, A.M., Schleimer, R.P., and Lichtenstein, L.M. (1987). Morphologic mast cell cycles. Cell. Immunol. *105*, 199–204.

80. McGettrick, A.F., and O'Neill, L.A.J. (2020). The Role of HIF in Immunity and Inflammation. Cell Metabol. *32*, 524–536.

81. Ando, T., Xiao, W., Gao, P., Namiranian, S., Matsumoto, K., Tomimori, Y., Hong, H., Yamashita, H., Kimura, M., Kashiwakura, J.I., et al. (2014). Critical role for mast cell Stat5 activity in skin inflammation. Cell Rep. *6*, 366–376.

82. Ikeda, K., Nakajima, H., Suzuki, K., Watanabe, N., Kagami, S.i., and Iwamoto, I. (2005). Stat5a is essential for the proliferation and survival of murine mast cells. Int. Arch. Allergy Immunol. *137* (*Suppl 1*), 45–50.

83. Reinhart, R., Rohner, L., Wicki, S., Fux, M., and Kaufmann, T. (2018). BH3 mimetics efficiently induce apoptosis in mouse basophils and mast cells. Cell Death Differ. *25*, 204–216.

84. Beghdadi, W., Madjene, L.C., Benhamou, M., Charles, N., Gautier, G., Launay, P., and Blank, U. (2011). Mast cells as cellular sensors in inflammation and immunity. Front. Immunol. *2*, 37.

85. Abraham, S.N., and St John, A.L. (2010). Mast cell-orchestrated immunity to pathogens. Nat. Rev. Immunol. *10*, 440–452.

86. Galli, S.J., Nakae, S., and Tsai, M. (2005). Mast cells in the development of adaptive immune responses. Nat. Immunol. *6*, 135–142.

87. Peschke, K., Weitzmann, A., Heger, K., Behrendt, R., Schubert, N., Scholten, J., Voehringer, D., Hartmann, K., Dudeck, A., Schmidt-Supprian, M., and Roers, A. (2014). IkappaB kinase 2 is essential for IgE-induced mast cell *de novo* cytokine production but not for degranulation. Cell Rep. *8*, 1300–1307.

88. Metcalfe, D.D., Baram, D., and Mekori, Y.A. (1997). Mast cells. Physiol. Rev. *77*, 1033–1079.

89. Choi, J.K., and Kim, S.H. (2014). Inhibitory effect of galangin on atopic dermatitis-like skin lesions. Food Chem. Toxicol. *68*, 135–141.

90. Choi, J.K., Jang, Y.H., Lee, S., Lee, S.R., Choi, Y.A., Jin, M., Choi, J.H., Park, J.H., Park, P.H., Choi, H., et al. (2017). Chrysin attenuates atopic dermatitis by suppressing inflammation of keratinocytes. Food Chem. Toxicol. *110*, 142–150.

91. Yu, J.H., Jin, M., Choi, Y.A., Jeong, N.H., Park, J.S., Shin, T.Y., and Kim, S.H. (2017). Suppressive effect of an aqueous extract of Diospyros kaki calyx on dust mite extract/2,4-dinitrochlorobenzene-induced atopic dermatitis-like skin lesions. Int. J. Mol. Med. *40*, 505–511.

92. Beck, L.A., Cork, M.J., Amagai, M., De Benedetto, A., Kabashima, K., Hamilton, J.D., and Rossi, A.B. (2022). Type 2 Inflammation Contributes to Skin Barrier Dysfunction in Atopic Dermatitis. JID Innov. *2*, 100131.

93. Gu, H., Kim, W.H., An, H.J., Kim, J.Y., Gwon, M.G., Han, S.M., Leem, J., and Park, K.K. (2018). Therapeutic effects of bee venom on experimental atopic dermatitis. Mol. Med. Rep. *18*, 3711–3718.

94. McAleer, M.A., and Irvine, A.D. (2013). The multifunctional role of filaggrin in allergic skin disease. J. Allergy Clin. Immunol. *131*, 280–291.

95. Gutowska-Owsiak, D., and Ogg, G.S. (2013). Cytokine regulation of the epidermal barrier. Clin. Exp. Allergy *43*, 586–598.

96. Hänel, K.H., Cornelissen, C., Lüscher, B., and Baron, J.M. (2013). Cytokines and the skin barrier. Int. J. Mol. Sci. *14*, 6720–6745.

97. Kim, B.E., and Leung, D.Y. (2012). Epidermal barrier in atopic dermatitis. Allergy Asthma Immunol. Res. *4*, 12–16.

98. Kim, B.E., and Leung, D.Y.M. (2018). Significance of Skin Barrier Dysfunction in Atopic Dermatitis. Allergy Asthma Immunol. Res. *10*, 207–215.

OMTN, Volume *34*

Supplemental information

The decoy oligodeoxynucleotide against HIF-1α and STAT5 ameliorates atopic dermatitis-like mouse model

Mi-Gyeong Gwon, Jaechan Leem, Hyun-Jin An, Hyemin Gu, Seongjae Bae, Jong Hyun Kim, and Kwan-Kyu Park

Supplemental Information

Figure S1

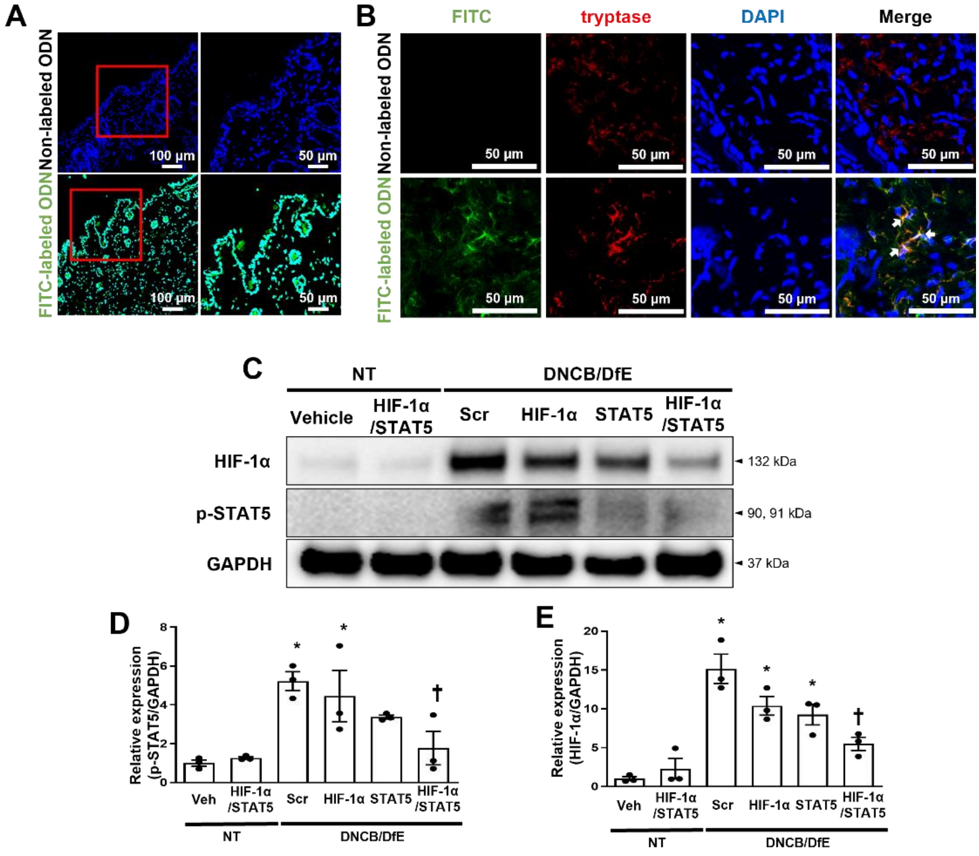

Figure S1. Introduction efficiency of the FITC-labeled HIF-1α/STAT5 decoy ODN in Balb/c mice and the inhibitory effect of the HIF-1α/STAT5 decoy ODN on HIF-1α and STAT5 expression.

(A) Fluorescence staining images show the transfection efficiency of the FITC-labeled HIF-1α/STAT5 decoy ODN (green) in the dorsal skin (n=2). The FITC-labeled decoy ODN was injected via the tail vein of the mice. The nuclei were stained with DAPI (blue). The right image of each group is an enlargement of the area marked with a red square. Scale bar = 100 or 50 μm. (B) Representative immunofluorescence staining image shows that the HIF-1α/STAT5 decoy ODN effectively enters mast cells in mouse skin (n=3). Green color indicates FITC-labeled decoy ODN, and red color indicates tryptase. The yellow signal in the merge panel is the co-localization of FITC-labeled decoy ODN and tryptase. The arrows indicate mast cells that show a double-positive signal of FITC-labeled decoy ODN and tryptase. (C) The protein expressions of HIF-1α and p-STAT5 were analyzed by immunoblotting. GAPDH was used to normalize the equal loading of all protein samples. The quantitative graphs show the protein expression of (D) HIF-1α and (E) p-STAT5 normalized with GAPDH. The bar graphs were quantified from three independent immunoblotting data. Vehicle (Veh): distilled water; Scrambled (Scr) ODN:

Scrambled decoy ODN; HIF-1α/STAT5 ODN: HIF-1α/STAT5 decoy ODN; NT: DNCB and DfE non-treated group; DNCB/DfE: DNCB and DfE sensitized group. *p < 0.05 compared with the vehicle group; †p < 0.05 compared with the DNCB/DfE-sensitized with Scr ODN group.

Figure S2

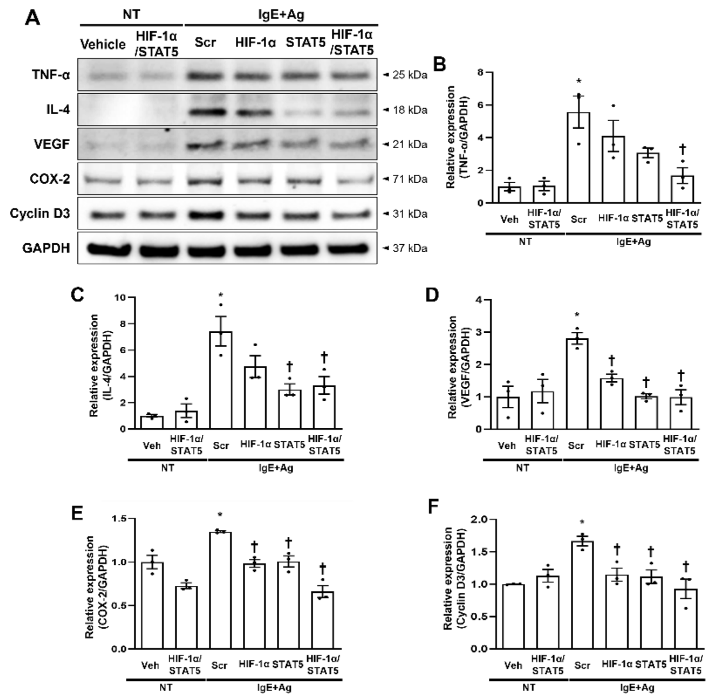

Figure S2. The inhibitory effect of the single decoy ODN and HIF-1α/STAT5 decoy ODN on HIF-1α and STAT5 downstream gene expression.

To investigate the effect of single decoy ODN, we designed two single decoy ODNs, a HIF-1α-scramble arm, and a STAT5-scramble arm. The RBL-2H3 cells were transfected with HIF-1α, STAT5, and HIF-1α/STAT5 decoy ODN (60 nM). Western blot analysis of cytokines, HIF-1α downstream genes (VEGF, COX-2), and STAT5 downstream gene (cyclin D3). GAPDH was used as a loading control. Quantification of immunoblots for (B) TNF-α, (C) IL-4, (D) VEGF, (E) COX-2, and (F) cyclin D3 after normalization with GAPDH. The graphs were quantified from three immunoblot data. Vehicle (Veh): distilled water; Scrambled (Scr) ODN: Scrambled decoy ODN; HIF-1α/STAT5 ODN: HIF-1α/STAT5 decoy ODN; NT: IgE+Ag non-treated; IgE+Ag: IgE+Ag treated. *p < 0.05 compared with the vehicle group; †p < 0.05 compared with the IgE+Ag-sensitized with Scr ODN group.

Figure S3

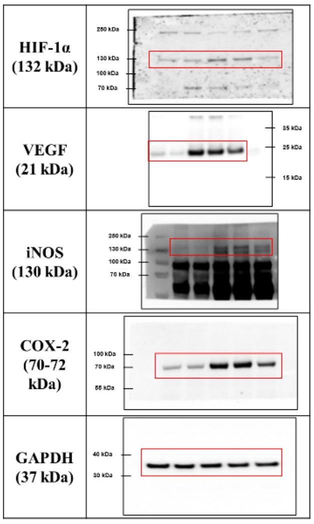

Figure 2C WB

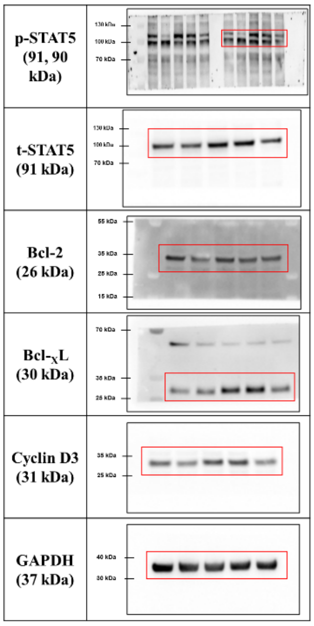

Figure 2H WB

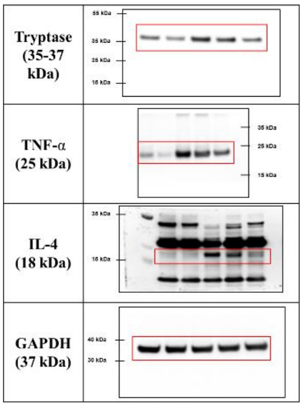

Figure 3C WB

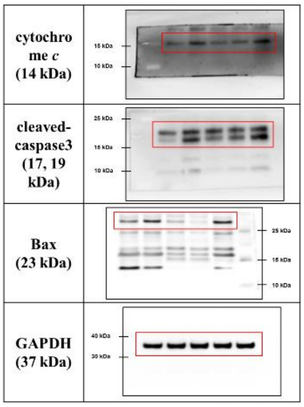

Figure 4B WB

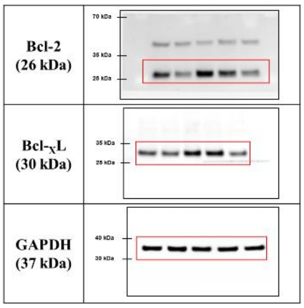

Figure 4F WB

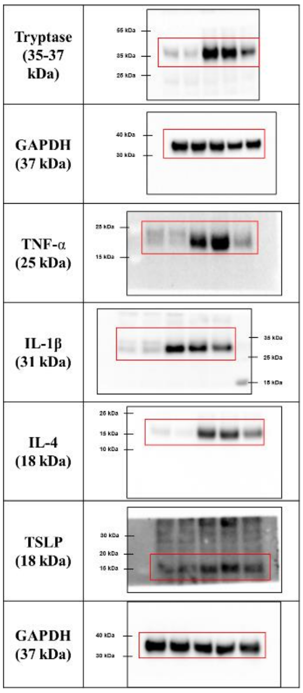

Figure 6C and 6E WB

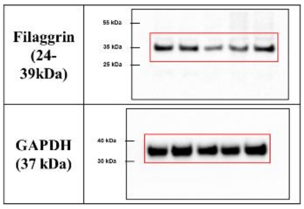

Figure 6L WB

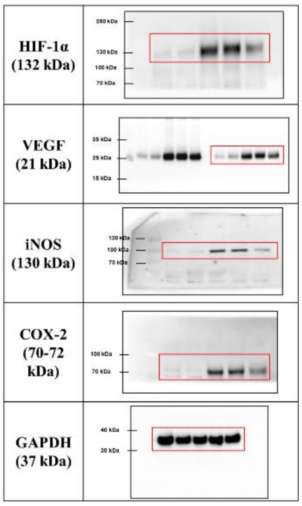

Figure 7C WB

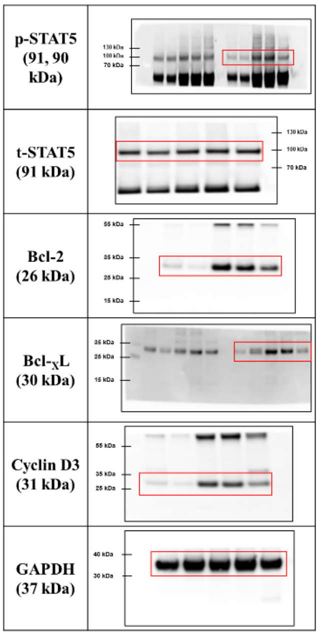

Figure 7H WB

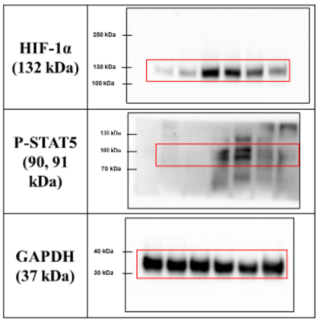

Figure S1C WB

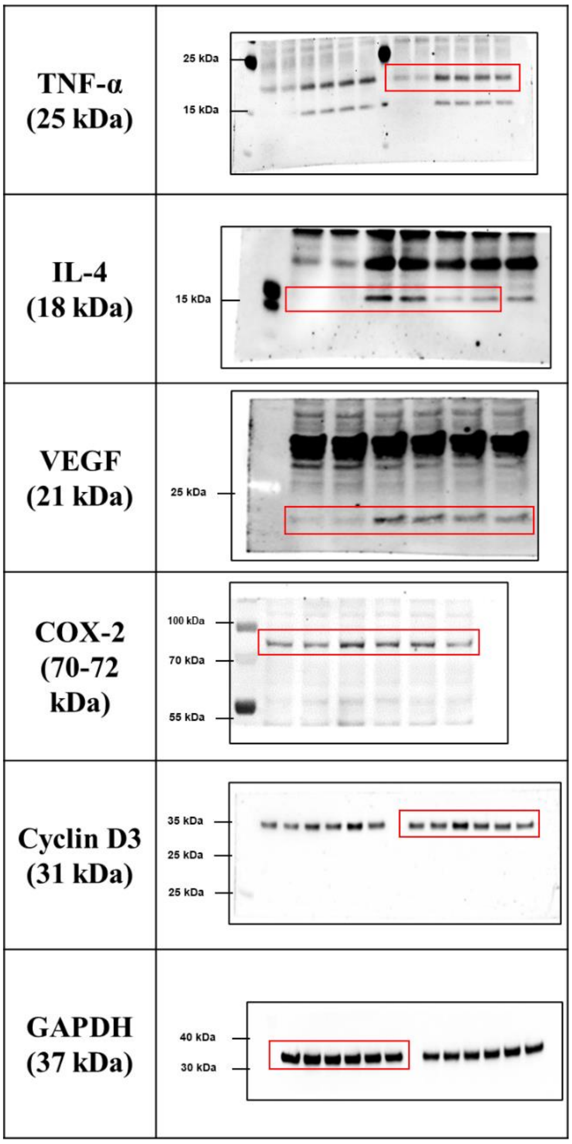

Figure S2A WB

Figure S3. Uncropped Western blot images from the blot are shown in all Figures.

The red boxes outline the areas presented in all the figures.
